# Spatially confined genome editing enables localized combination immunotherapy

**DOI:** 10.64898/2026.01.14.699490

**Published:** 2026-02-18

**Authors:** Xiaoyue Yang, Laura Tong, Yidan Pan, Jin Huang, Zhongchao Yi, Daheng He, Jingpeng Liu, Chi Wang, Ying Liang, Sheng Tong

**Affiliations:** 1 F. Joseph Halcomb III, M.D. Department of Biomedical Engineering, University of Kentucky, Lexington, Kentucky 40536, USA; 2 Department of Bioengineering, Stanford University, Stanford, California 94305, USA; 3 Department of Genetics, The University of Texas MD Anderson Cancer Center, Houston, Texas 77030, USA; 4 Division of Cancer Biostatistics, Department of Internal Medicine, University of Kentucky, Lexington, Kentucky 40536, USA; 5 New York Blood Center, New York, New York 10065, USA

## Abstract

Immune checkpoint blockade can elicit durable antitumor responses, yet tumor heterogeneity and adaptive resistance often necessitate combination strategies that increase systemic toxicity. In vivo genome editing offers a programmable route to durable immunomodulation but remains difficult to spatially confine in solid tumors. Here we develop a magnetically gated genome-editing platform that enables spatially confined immunomodulation. The system integrates a non-replicating baculoviral vector with magnetic nanoparticles (MBV), in which magnetic activation restores viral transduction despite complement-mediated inactivation, thereby confining CRISPR activity to tumor regions. Baculoviral transduction engages antiviral innate programs that promote chemokine signaling and antigen presentation in tumors. In a syngeneic colon cancer model, MBV-mediated disruption of *Pdl1* restricts checkpoint loss to tumor tissue while preserving immune activation, enhancing immune infiltration and suppressing tumor growth. Local *Pdl1* editing synergizes with CTLA-4 blockade, extending survival without overt toxicity. These findings define MBV as a controllable genome-editing architecture for localized combination immunotherapy.

Immune checkpoint blockade can elicit durable antitumor responses, yet tumor heterogeneity and adaptive resistance frequently necessitate combination strategies that are constrained by mismatched pharmacokinetics and systemic immune activation^1–7^. Approaches that enable localized modulation of immunosuppressive pathways within tumors could expand the therapeutic window of combination immunotherapy. In vivo genome editing offers a programmable and durable means of disrupting immune checkpoints^8–10^; however, once administered, current viral and non-viral delivery systems provide limited spatial control, making it difficult to confine editing to tumor regions while avoiding unintended editing in normal tissues and off-target immune perturbation^11–15^.

Baculovirus represents a programmable delivery backbone with properties that are advantageous yet underexplored in mammalian systems. Although replication-defective in mammalian cells, baculoviral vectors support large genetic payloads and efficient nuclear delivery, enabling multiplexed CRISPR-mediated genome editing^16–18^. Upon in vivo administration, baculoviral particles are rapidly inactivated by complement, restricting systemic persistence and limiting efficient transduction^19^. Concurrently, unmethylated CpG motifs within the baculoviral genome engage innate immune sensors and induce interferon and chemokine programs that reshape local immune environments^20, 21^. Antiviral signaling enhances immune cell recruitment but also induces compensatory checkpoint upregulation, including PD-L1 expression^22–24^, creating a tension between antiviral immune activation and checkpoint-mediated immune suppression.

To impose spatial regulation on genome editing, we integrate magnetic nanoparticles with a non-replicating baculoviral vector to generate a magnetically activatable baculovirus (MBV), extending our previous demonstration of magnetically controlled viral delivery to enable spatially confined genome editing^25^. Magnetic activation functions as a physical on-switch that enhances local viral transduction despite complement-mediated inactivation, thereby confining viral delivery and subsequent CRISPR activity to magnetically targeted tumor regions. Outside the activated region, complement-mediated inactivation limits dissemination, establishing orthogonal physical and biological constraints over vector activity in vivo. This architecture enables spatially restricted genome editing while preserving virus-induced innate immune amplification.

We apply this platform to disrupt *Pdl1* within solid tumors as a stringent in vivo test. In a syngeneic colon cancer model with limited responsiveness to PD-L1 antibody blockade, magnetic activation enables localized CRISPR-mediated checkpoint disruption without detectable editing in adjacent normal tissues. In addition to checkpoint disruption, MBV transduction induces chemokine signaling, antigen-presentation pathways and interferon responses that reshape the tumor immune microenvironment. These coordinated effects are associated with enhanced immune infiltration, effector activation and suppression of tumor growth, resulting in prolonged survival. Local *Pdl1* editing synergizes with systemic CTLA-4 blockade without overt toxicity. Together, these findings establish MBV as a controllable genome-editing technology that integrates physical targeting, biological containment, and programmable immunomodulation for localized combination cancer immunotherapy.

## Results

### Magnetic activation restores baculoviral transduction under complement constraint

**Fig. 2.**
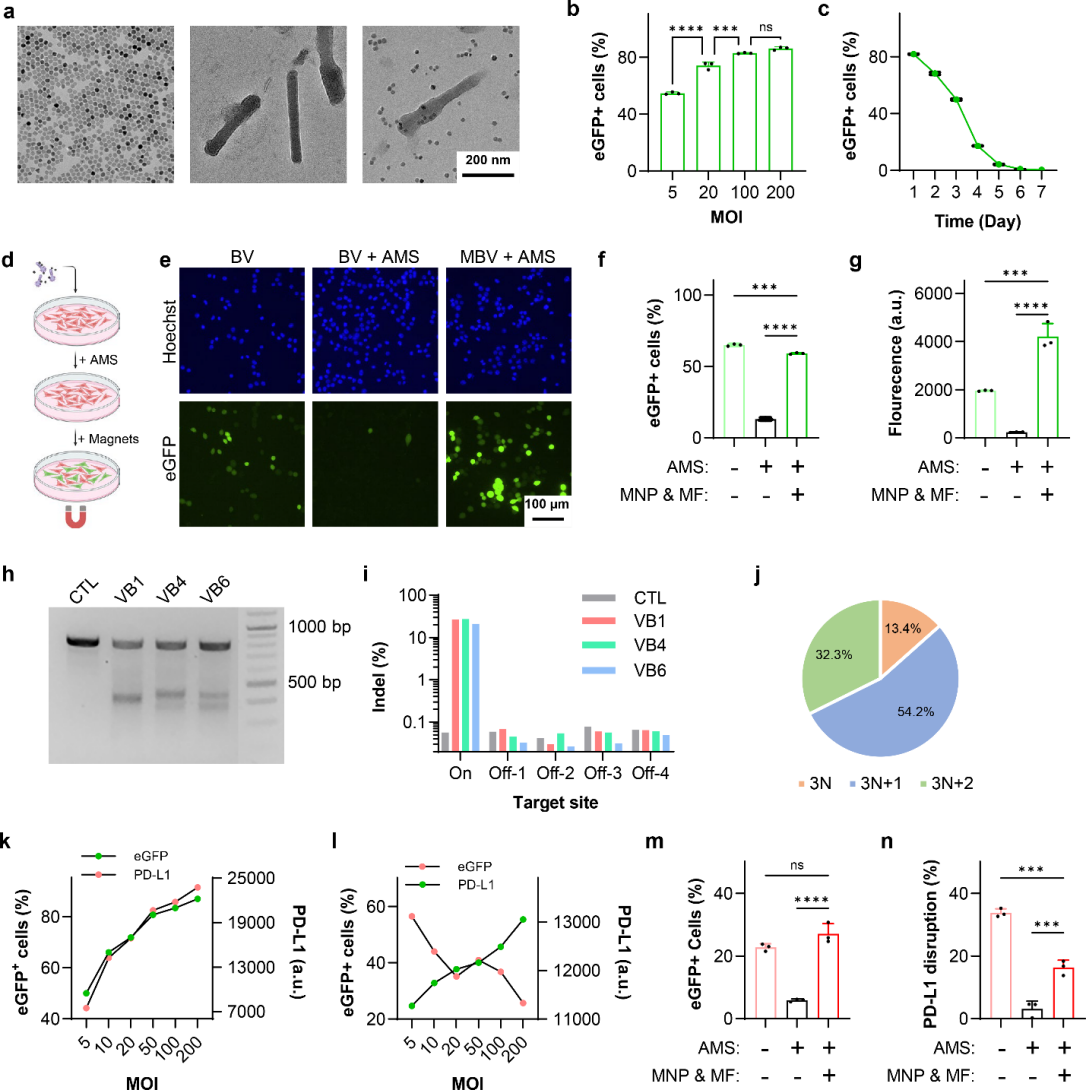
Characterization of MBV. **a**. Transmission electron microscopy images of magnetic nanoparticles (MNPs), baculovirus (BV), and magnetically modified baculovirus (MBV). Samples in the two right panels were negatively stained with phosphotungstic acid. **b**, **c**. Efficiency and temporal profile of BV transduction in MC38 cells. **d.** Schematic of the in vitro assay used to evaluate magnetic activation of MBV-eGFP under complement pressure. **e**. Representative fluorescence microscopy images of MC38 cells incubated with BV-eGFP alone, BV-eGFP in the presence of adult mouse serum (AMS), and MBV-eGFP with AMS and magnetic activation. **f**, **g**. Flow cytometry analysis of AMS-mediated inhibition and magnetic activation of BV-eGFP-driven eGFP expression. **h**. T7 endonuclease I (T7E1) analysis of three candidate guide RNAs (gRNAs; VB1, VB4, and VB6) targeting exon 3. **i**. Evaluation of on- and off-target activities of the three candidate gRNAs by next generation sequencing (NGS). Data represent the mean of three technical replicates. **j**. Distribution of VB1-induced insertion and deletion (Indel) events categorized by resulting reading-frame shifts, quantified by NGS. **k**, **l.** Flow cytometry analysis of eGFP and PD-L1 expression in MC38 cells following transduction by BV-eGFP and BV-*Pdl1*-eGFP, respectively. **m**, **n**. Flow cytometry analysis of AMS inhibition and magnetic activation of BV-*Pdl1*-eGFP-mediated eGFP expression and PD-L1 disruption in MC38 cells. Data are presented as mean ± s.d. ns, ***, and **** denote not significant, *P* < 0.001, and *P* < 0.0001, respectively.

We first examined whether magnetic activation could overcome complement-mediated restriction of baculoviral transduction. MBV was generated by electrostatic conjugation of MNPs to the surface of baculoviral vectors (BVs). MNPs functionalized with TAT peptides were synthesized using a two-step procedure that enabled control over particle size, dispersity, and peptide density (Fig. 2a, Supplementary Fig. S1)^26^. The cationic TAT peptides facilitated binding to the negatively charged baculoviral envelope. BV vectors encoding SpCas9, reporters, and gRNAs were constructed within a single viral genome, leveraging the large cargo capacity of BV to improve editing efficiency and reduce production complexity (Supporting Fig. S2). MBV complexes were assembled by mixing MNPs with BV at a ratio of ~3 μg Fe per 10^7^ pfu for 10 min in PBS (Fig. 2a), a condition optimized to preserve gp64-mediated endosomal escape while ensuring stable nanoparticle association.

BV transduction efficiency was first evaluated in MC38 murine colon adenocarcinoma cells, the cell line used to establish the syngeneic tumor model. More than 50 % of cells expressed eGFP at a multiplicity of infection (MOI) of 5 (Fig. 2b). eGFP expression peaked at 24 h and declined to undetectable levels by day 7 (Fig. 2c), consistent with transient BV-mediated transgene expression.

To assess complement inhibition and magnetic activation as functional off- and on-switches in MC38 cells, BV transduction was evaluated in the presence of 50% adult mouse serum (AMS), which contains a fully developed complement system (Fig. 2d, Supplementary Fig. S3). AMS markedly reduced both the fraction of eGFP-positive cells and expression intensity (Fig. 2e). In contrast, magnetic activation of MBV restored robust transduction under complement pressure, yielding higher expression levels than BV in AMS-free conditions. Flow cytometry showed ~80% inhibition of BV transduction by AMS, whereas magnetically activated MBV fully rescues transgene expression (Fig. 2f, g). These results confirm that complement sensitivity suppresses off-target BV activity, while magnetic activation enables localized gene delivery.

The murine *Pdl1* locus comprises five exons on chromosome 19. Six candidate guide RNAs targeting early exons or key structural regions were designed and screened in MC38 cells (Supplementary Table S1)^27^. Based on T7E1 cleavage and next generation sequencing (NGS) analysis, a guide targeting exon 3 was selected (Fig. 2h, i, Supplementary Fig. S4). NGS revealed that ~87% of Cas9-induced indels were frameshift mutations, indicating efficient functional disruption (Fig. 2j).

BV transduction increased PD-L1 expression in MC38 cells in a transduction-dependent manner, consistent with interferon-mediated checkpoint induction (Fig. 2k)^22^. In contrast, BV-*Pdl1* reduced PD-L1 expression at matched MOIs without inducing high cytotoxicity (Fig. 2l, Supplementary Fig. S5). MBV-*Pdl1* achieved higher disruption efficiency than BV-*Pdl1* (Supplementary Fig. S6). AMS suppressed both eGFP expression and *Pdl1* disruption by BV-*Pdl1*, whereas magnetic activation of MBV-*Pdl1* partially restored editing activity (Fig. 2m, n). These findings establish complement inactivation as a biological containment mechanism and magnetic activation as a physical rescue mechanism, forming orthogonal constraints that regulate vector activity.

### BV transduction induces immune-associated signaling in vitro

To isolate immune effects attributable to BV transduction independent of *Pdl1* disruption, a control vector encoding Cas9 without a targeting guide RNA (BV-Cas9) was generated. Similar to BV-eGFP, BV-Cas9 increased PD-L1 expression relative to untreated cells, whereas BV-*Pdl1* disrupted PD-L1 expression in 45.5% of cells (Supplementary Fig. S7).

**Fig 3.**
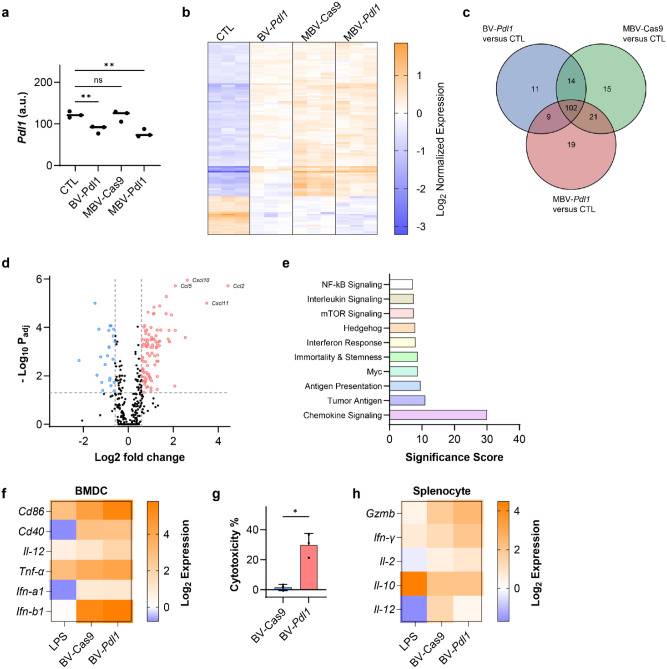
BV-*Pdl1* enhances anticancer immunity through coordinated antiviral responses and *Pdl1* disruption. Transcriptomic responses of MC38 cells treated with BV-*Pdl1*, MBV-Cas9, and MBV-*Pdl1* were analyzed using the nCounter^®^ Tumor Signaling 360 Panel. **a**. *Pdl1* expression in MC38 cells following the indicated treatments. ns and ** denote no significance and *P* < 0.01, respectively. **b**. Heat map of normalized gene expression across treatment groups. n = 3 per group. **c**. Venn diagram of differentially expressed genes (|log_2_FC| ≥ 1, *P*_adj_ < 0.05) in BV-*Pdl1*-, MBV-Cas9-, MBV-*Pdl1*-treated cells relative to untreated controls. **d**. Differential gene expression in MC38 cells treated with MBV-*Pdl1* relative to PBS control. *P* values were adjusted using the Benjamini and Yekutieli method for multiple testing correction. **e**. Pathway enrichment analysis of MBV-*Pdl1*-treated MC38 cells relative to PBS control. Bone marrow-derived dendritic cells (BMDCs) were cocultured with BV-*Pdl1*-treated MC38 cells. **f**. Gene expression in BMDCs quantified by RT-qPCR and normalized to coculture with untreated MC38 cells. Lipopolysaccharide (LPS) treatment were used as a positive control (n = 3 per group). Splenocytes were cocultured with BV-*Pdl1*-treated MC38 cells. **g**. Splenocytes-mediated cytotoxicity against BV-transduced MC38 cells relative to untreated control. Data are presented as mean ± s.d. * denotes *P* < 0.05. **h**. Gene expression in splenocytes quantified by RT-qPCR and normalized to coculture with untreated MC38 cells. LPS treatment were used as a positive control (n = 3 per group).

Transcriptomic profiling of MC38 cells treated with BV-*Pdl1*, MBV-Cas9, and MBV-*Pdl1* was performed using the nCounter^®^ Tumor Signaling 360 Panel. Both BV-*Pdl1* and MBV-*Pdl1* reduced *Pdl1* transcript level (Fig. 3a). In contrast, global transcriptional changes were largely shared across all treated groups and distinct from untreated controls, indicating that BV transduction was the dominant driver of immune-related gene expression (Fig. 3b, c).

BV-treated cells exhibited marked upregulation of chemokines associated with lymphocyte recruitment, including *Ccl2*, *Cxcl11*, *Cxcl10*, *Ccl5* (Fig. 3d, Supplementary Fig. S8a, c). Gene-set analysis revealed activation of chemokine signaling, antigen processing and presentation, and interferon response pathways (Fig. 3e, Supplementary Fig. S8b, d), consistent with a coordinated antiviral response.

To evaluate downstream immune activation, BV-transduced MC38 cells were cocultured with bone marrow-derived dendritic cells (BMDCs). Coculture induced upregulation of canonical maturation markers (*Cd86*, *Cd40*, *Il-12*, *Tnf-*α, *Ifn-*α*1*, and *Ifn-*β*1*), indicating dendritic cell activation (Fig. 3f). When cocultured with splenocytes, BV-*Pdl1*-treated MC38 cells elicited greater cytotoxicity than BV-Cas9-treated cells (Fig. 3g). Correspondingly, splenocytes exposed to BV-*Pdl1*-treated cells exhibited elevated expression of cytotoxic effector genes, including *Gzmb*, *Ifn-*γ, relative to BV-Cas9 (Fig. 3h). Disruption of *Pdl1* mitigates antiviral-induced compensatory PD-L1 upregulation, coupling immune recruitment with checkpoint inhibition to sustain cytotoxic effector activity.

### Magnetic gating enables spatially confined genome editing in vivo

**Fig. 4.**
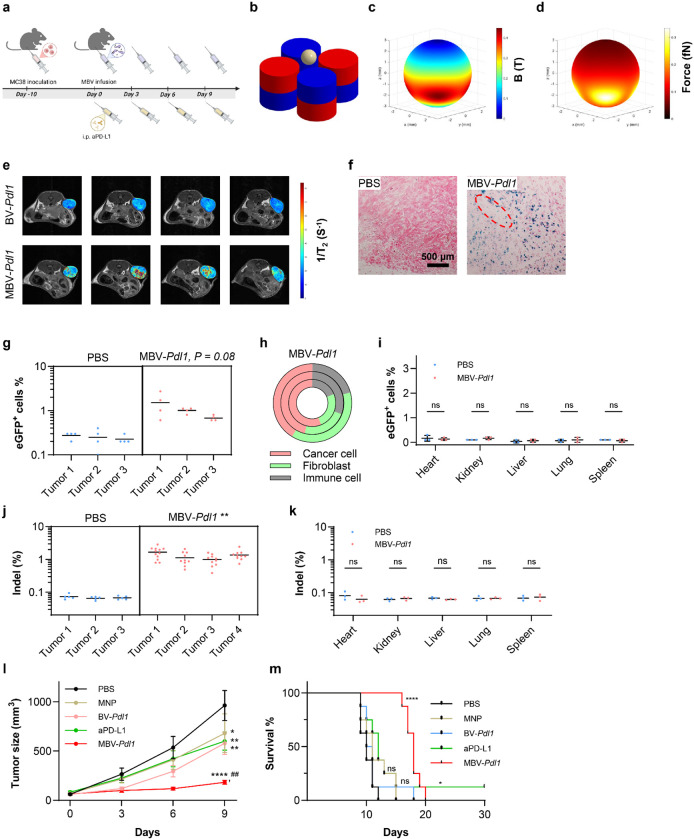
Magnetic gating enables spatially confined genome editing in vivo. **a**. Schematic of the treatment timeline. **b**. Schematic of intratumoral magnetic activation of MBV. The dimensions and relative positions of the magnets and tumor are drawn to scale. **c**, **d**. Simulated distributions of magnetic flux density (**c**) and magnetic force (**d**) on the tumor surface. Distribution of MBV inside the tumor was assessed by MRI and histological staining. **e**. Representative consecutive MRI cross sections overlaid with computed T_2_ relaxivity maps of the tumor. **f**. Representative histological images of tumors treated with PBS or MBV-*Pdl1*. Tumor sections were stained for MNPs by Prussian blue and counterstained with nuclear red. The dashed red circle denotes the needle track. In vivo transduction by MBV was evaluated using MBV-*Pdl1* carrying an eGFP reporter. **g**. Flow cytometry quantification of eGFP^+^ cells in tumors treated with PBS or MBV-*Pdl1*. Fragment-level measurements are shown to illustrate intratumoral heterogeneity; statistical comparisons were performed using mouse-level means. **h**. Relative abundance of CD44^+^ tumor cells, ER-TR7^+^ fibroblasts, and CD45^+^ immune cells among eGFP^+^ cells is shown for each treated mouse. PBS controls are not shown because eGFP^+^ events were at background levels, precluding meaningful compositional analysis. **i**. Flow cytometry quantification of eGFP^+^ cells in major organs following PBS or MBV-*Pdl1* treatment. **j**. Next-generation sequencing (NGS) quantification of indel frequency in tumors following PBS or MBV-*Pdl1* treatment. **k**. NGS quantification of indel frequency in major organs following PBS or MBV-*Pdl1* treatment. **l**. Tumor volume measured from treatment initiation. Data are presented as mean ± s.e.m. **m**. Kaplan-Meier survival curves following the indicated treatment (n = 8 per group). ns, *, **, ***, and **** denote no significance, *P* < 0.05, *P* < 0.01, *P* < 0.001, and *P* < 0.0001 versus PBS, respectively; ^##^ denote *P* < 0.01 versus anti-PD-L1.

To evaluate the effect of localized *Pdl1* disruption in vivo, C57BL/6 mice bearing established MC38 tumors (~50 mm^3^) were treated by intratumoral infusion of MBV-*Pdl1* followed by magnetic activation (Fig. 4a). A custom magnetic array composed of four cylindrical NdFeB magnets arranged in alternating polarity was integrated into a 3D-printed animal bed to ensure reproducible tumor positioning (Fig. 4b, Supplementary Fig. S9). Mice were maintained under anesthesia with the tumor aligned within the magnetic field for 1 h after each infusion. Simulated magnetic flux density and force distribution at the tumor surface were comparable to in vitro activation conditions (Fig. 4c, d, Supplementary Fig. S10).

Magnetic nanoparticles enabled noninvasive tracking of MBV distribution by T_2_-weighted MRI (Supplementary Fig. S11). MRI imaging and histological analysis revealed localized accumulation of MBV primarily surrounding the injection track (Fig. 4e, f), similar to other locally administered large nanoparticles^28^. To quantify intratumoral transduction, tumors treated with MBV-*Pdl1* were divided into four fragments and analyzed by flow cytometry. eGFP^+^ cells ranged from 0.6% to 2.7% across fragments, reflecting heterogeneous intratumoral distribution (Fig. 4g). The majority of eGFP^+^ cells were CD44^+^ tumor cells with lower levels detected in ER-TR7^+^ fibroblasts, and CD45^+^ immune cells (Fig. 4h). It should be noted that BV has low transduction efficiency in cells of hematopoietic origin^29^. The eGFP signals in CD45^+^ cells likely reflect the rapid clearance of MBV transduced tumor cells. No eGFP expression was detected in major organs, and ex vivo fluorescence imaging further confirmed localization to treated tumors (Fig. 4i, Supplementary Fig. S12). Next-generation sequencing confirmed *Pdl1* indels in tumors but not in distal organs following MBV-*Pdl1* treatment (Fig. 4j, k). These data indicate that magnetic activation confines viral transduction and subsequent CRISPR activity to targeted tumor sites while complement-mediated inactivation limits systemic dissemination.

Therapeutic efficacy was evaluated by comparing PBS, MNP alone, BV-*Pdl1*, MBV-*Pdl1*, and systemic anti-PD-L1 antibody (aPD-L1). MBV-*Pdl1* was administered every three days for four doses to account for heterogeneous intratumoral distribution and transient transgene expression (Fig. 4a). MBV-*Pdl1* produced the strongest tumor growth suppression among all groups, including aPD-L1, whereas MNP or BV-*Pdl1* alone induced only modest delay (Fig. 4l). MBV-*Pdl1* significantly prolonged survival, with a median survival of 18 days compared with 10 days for PBS and 12 days for aPD-L1 (Fig. 4m). No significant body weight loss was observed (Supplementary Fig. 13).

Dose escalation of MBV-*Pdl1* did not further improve survival relative to standard dosing but remained superior to MBV-eGFP (Supplementary Fig. S14). Tumors derived from MC38 PD-L1 knockout cells exhibited only marginally reduced growth compared with wild-type tumors (Supplementary Fig. S15), indicating that tumor-intrinsic PD-L1 loss alone is insufficient to account for the observed therapeutic effect.

### MBV-mediated disruption of *Pdl1* prevents compensatory checkpoint signaling and enhances immune infiltration

**Fig 5.**
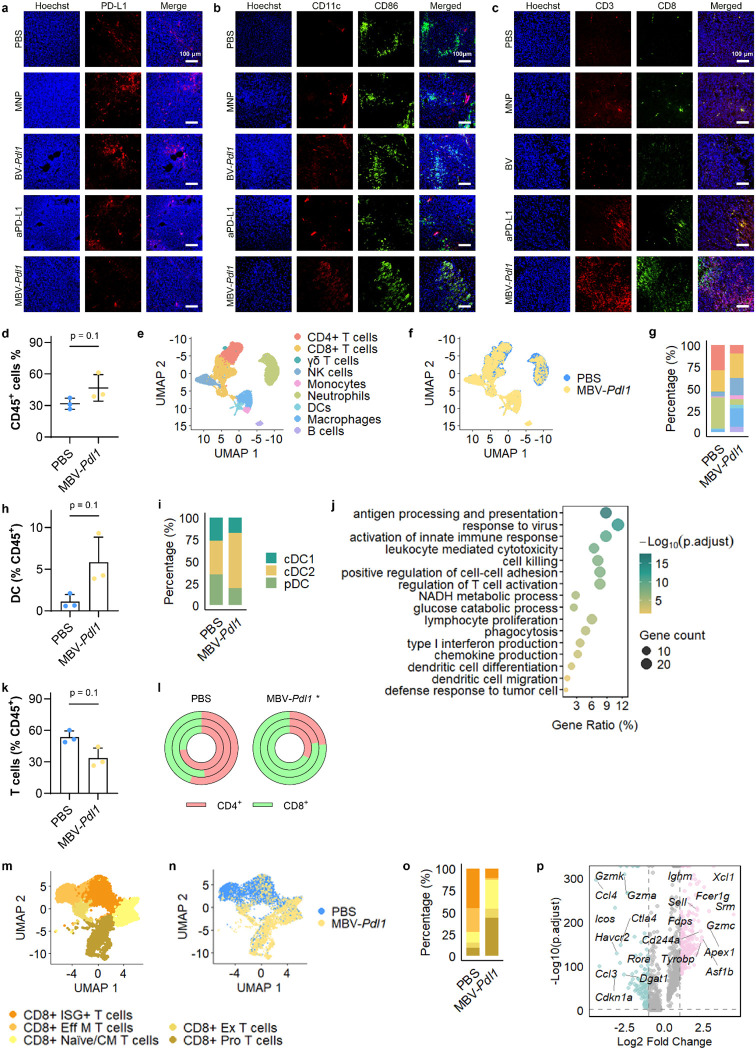
MBV-*Pdl1* modulates the tumor immune microenvironment. **a**, **b**, **c**. Representative fluorescence microscopy images of tumors stained for PD-L1 (**a**), activated dendritic cells (CD11C^+^CD86^+^); **b**), and CD8 T cells (CD3^+^CD8^+^); **c**) following treatment with PBS, MNP, BV-*Pdl1*, anti-PD-L1, or MBV-*Pdl1*. Nuclei were counterstained with Hoechst. **d**. Flow cytometry quantification of CD45^+^ cells in tumors treated with PBS or MBV-*Pdl1*. The effects of MBV-*Pdl1* treatment on the tumor immune microenvironment were further analyzed by single-cell RNA sequencing. **e**. UMAP embedding showing clustering of tumor-infiltrating immune cells. **f**. UMAP showing the distribution of immune cells in PBS- and MBV-*Pdl1*-treated tumors. **g**. Proportions of major immune cell types in PBS- and MBV-*Pdl1*-treated tumors. **h**. Fraction of dendritic cells (DCs) among CD45^+^ cells in PBS or MBV-*Pdl1*-treated tumors. **i**. Proportions of DC subtypes in PBS- and MBV-*Pdl1*-treated tumors. **j**. Pathway enrichment analysis of DCs in MBV-*Pdl1*-treated tumor relative to PBS. **K**. Fraction of T (γδ, CD4^+^, and CD8^+^) cells among CD45^+^ cells in PBS or MBV-*Pdl1*-treated tumors. **l**. Proportions of CD4^+^ and CD8^+^ T cells in PBS and MBV-*Pdl1*-treated tumors; each circle represents one mouse. **m**. UMAP embedding showing clustering of CD8^+^ T cells. **n**. UMAP showing the distribution of CD8^+^ T cells in PBS- and MBV-*Pdl1*-treated tumors. **o**. Proportions of CD8^+^ T cell subtypes in PBS- and MBV-*Pdl1*-treated tumors. **p**. Volcano plot of differentially expressed genes in CD8^+^ T cells from MBV-*Pdl1*-tumors relative to PBS. Cell population frequencies were compared across mice using a two-sided Mann–Whitney U test (n = 3 mice per group). Additional analyses are provided in Supplementary Fig. S17.

Immunostaining confirmed reduced PD-L1 expression in MBV-*Pdl1*-treated tumors but not in BV-*Pdl1*-treated tumors (Fig 5a). Both BV-*Pdl1* and MBV-*Pdl1* treatments increased infiltration of CD11c^+^CD86^+^ dendritic cells (Fig. 5b), whereas MBV-*Pdl1* and aPD-L1 treatment increased CD3^+^CD8^+^ T cell infiltration (Fig. 5c).

To comprehensively characterize the impact of MBV-*Pdl1* on the tumor immune microenvironment, we analyzed CD45^+^ immune cells isolated from tumors treated with PBS or MBV-*Pdl1*. Flow cytometry analysis revealed a near doubling of CD45^+^ immune cells in MBV-*Pdl1*-treated tumors relative to PBS controls (Fig. 5d). Single-cell RNA sequencing of tumor-infiltrating immune cells demonstrated marked shift in immune composition following MBV-*Pdl1* treatment (Fig. 5e–g, Supplementary Fig. S16). Tumors treated with PBS were dominated by three major populations – neutrophils, CD4^+^ T cells, and CD8^+^ T cells. MBV-*Pdl1* treatment increased immune diversity, with elevated proportions of macrophages, dendritic cells, B cells, natural killer (NK) cells, accompanied by reduced fractions of neutrophils, and CD4^+^ T cells.

Antigen-presenting cells, including macrophages, dendritic cells, and B cells, were substantially enriched following MBV-*Pdl1* treatment (Fig. 5g). Dendritic cells increased more than fourfold among CD45^+^ cells (Fig. 5h), with conventional type 2 dendritic cells (cDC2s) comprising the dominant subset (Fig. 5i, Supplementary Fig. S17a, b). Gene-expression analysis of tumor-infiltrating dendritic cells revealed enrichment of pathways associated with antiviral defense and antitumor immunity, including antigen processing and presentation, activation of innate immune responses, regulation of T cell activation, and type I interferon production (Fig. 5j).

Although the overall proportion of T cells decreased slightly, the CD8^+^/CD4^+^ T cell ratio increased significantly in MBV-*Pdl1*-treated tumors (Fig. 5k, l). Subclustering of CD8^+^ T cells further revealed a shift from effector and exhausted phenotypes toward naïve and cycling states (Fig. 5m–o). PBS-treated tumors contained higher proportions of effector and exhausted CD8^+^ T cells, whereas MBV-*Pdl1*-treated tumors exhibited a marked increase in naïve and cycling CD8^+^ T cells. Differential expression analysis revealed upregulation of genes associated with proliferation and cytotoxic potential, including *Xcl1, Gzmc*, and *Cd244a*, alongside reduced expression of inhibitory receptors (*Ctla4*, *Icos*, and *Havcr2*) (Fig. 5p). CD4^+^ T cell analysis revealed that MBV-*Pdl1* treatment increased memory and regulatory T cells fractions (Supplementary Fig. S17d–f). NK cells were also enriched following MBV-*Pdl1* treatment (Fig. 5g). Analysis of exhaustion and inhibitory markers (*Ctla4*, *Cd274*, and *Satb1*) alongside proliferation-associated genes (*Stmn1*, *Mcm3*, *Mcm5*) and activation-associated genes (*Gzmc*, *Ifitm2*, *Apex1*, *C1qbp*) revealed that NK cells in MBV-*Pdl1*-treated tumors exhibited transcriptional signatures consistent with antiviral activation and cytotoxic effector programs (Supplementary Fig. S17c).

### Localized MBV-*Pdl1* activation synergizes with systemic CTLA-4 blockade

**Fig 6.**
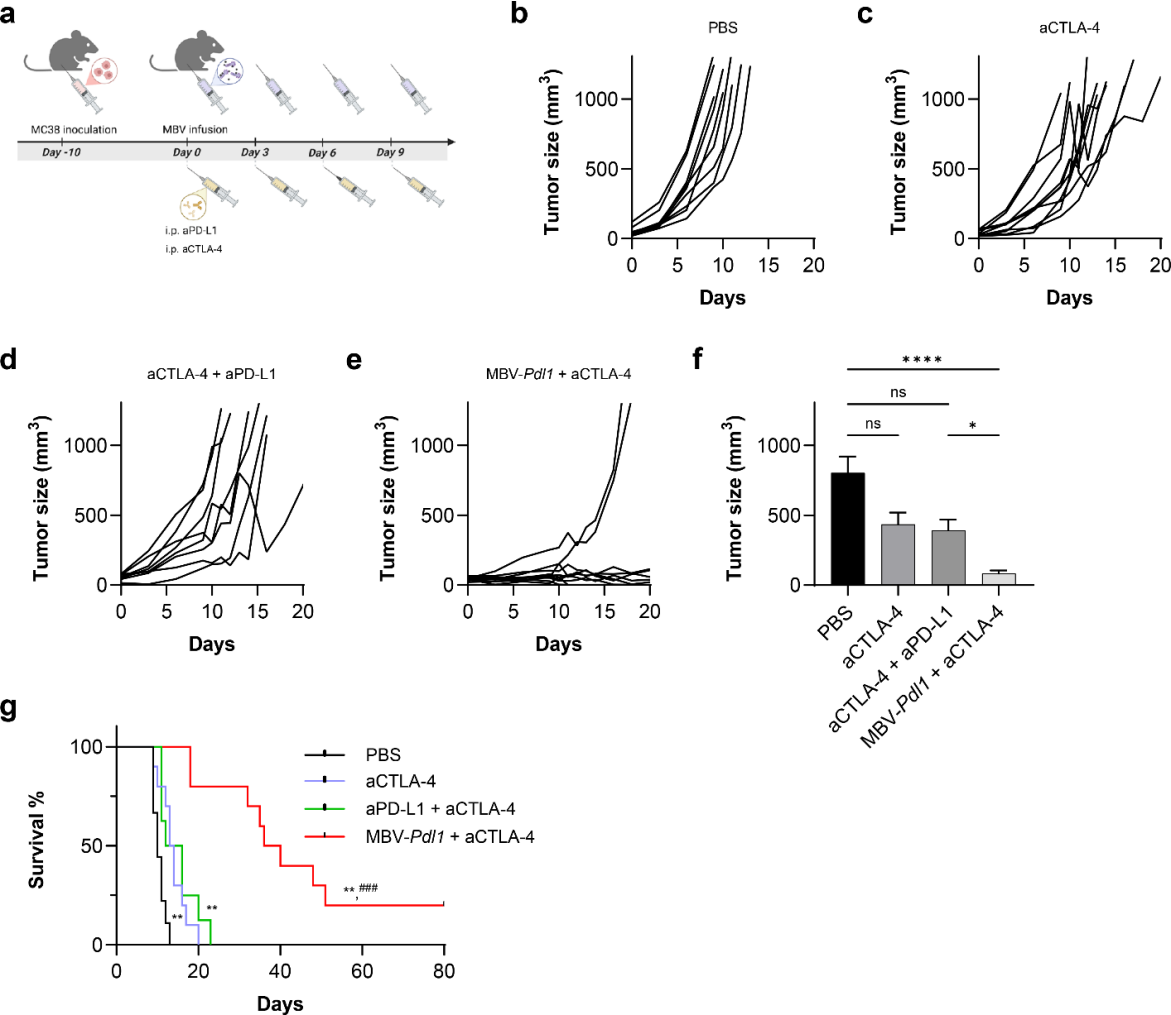
MBV-*Pdl1* and aCTLA-4 synergistically suppress tumor growth. **a**. Schematic of the treatment timeline. **b-e**, Tumor size measured from the initiation of treatment. Data are presented as mean ± s.e.m. **f**. Tumor size measured on day 9 after the initiation of treatment. Data are presented as mean ± s.e.m. ns, *, and **** denote no significance, *P* < 0.05, and *P* < 0.0001, respectively. **g**. Corresponding Kaplan-Meier survival curves following the indicated treatment. ** denotes *P* < 0.01 versus PBS; ^###^ denotes *P* < 0.001 versus anti-PD-L1 + anti-CTLA-4.

To assess compatibility with systemic immune checkpoint blockade, MBV-*Pdl1* was combined with anti-CTLA-4 antibody (aCTLA-4) in MC38 tumor-bearing mice (Fig. 6a). CTLA-4 blockade is a clinically relevant partner for PD1/PD-L1-targeted therapies and can reduce tumor-infiltrating regulatory T cells and attenuate their immunosuppressive activities^30^, a feature observed in MBV-*Pdl1* treated tumors. The combination produced greater tumor suppression than either monotherapy or antibody combinations (Fig. 6b–f). Median survival increased to 38 days in the MBV-*Pdl1* + aCTLA-4 group compared with 10 days for PBS, 13.5 days for aCTLA-4 alone, and 14 days for aCTLA-4 + aPD-L1 (Fig. 6g). Two mice in the combination group achieved complete tumor regression. No significant body-weight loss was observed, indicating favorable tolerability (Supplementary Fig. S18).

## Discussion

In vivo genome editing provides a programmable approach to tumor immunomodulation, yet strategies for regulating its spatial distribution within solid tissues remain limited. Systemic delivery of gene-editing agents risks unintended editing and immune perturbation outside the target region. Here, we integrate external physical regulation with intrinsic viral immune signaling to achieve spatially confined genome editing and localized immunomodulation within tumors.

We developed a hybrid genome-editing platform that decouples viral immunogenicity from replication by combining a non-replicating baculoviral vector with magnetic nanoparticles. A defining feature of MBV is the integration of orthogonal physical and biological constraints to regulate vector activity in vivo. Complement sensitivity suppresses off-target viral transduction, whereas magnetic activation concentrates vector-cell interactions and restores intracellular delivery at the target site. This coupling of an engineered physical on-switch with an endogenous biological off-switch confines viral entry, transgene expression, and CRISPR activity to magnetically targeted tumor regions while limiting dissemination and prolonged nuclease exposure. Transgene expression remains transient, whereas genome editing is durable, enabling sustained checkpoint disruption without persistent vector presence.

Beyond delivery control, baculovirus transduction intrinsically engages antiviral signaling pathways, including interferon responses, chemokine secretion, and antigen-presentation programs that promote immune-cell recruitment and priming. However, antiviral signaling also induces compensatory PD-L1 upregulation that can restrain effector function. Local disruption of *Pdl1* resolves this inhibitory feedback while preserving the immunostimulatory context generated by viral sensing. The limited efficacy of tumor-intrinsic PD-L1 knockout alone indicates that therapeutic benefit arises from coordinated antiviral immune activation and spatially confined genome editing rather than checkpoint disruption in isolation.

At the tissue level, MBV-*Pdl1* increases immune-cell density and diversity, particularly among antigen-presenting populations. Expansion of dendritic cells and enrichment of antigen-processing pathways are consistent with sustained T-cell priming within tumors. Transcriptional profiling supports recruitment and activation of effector populations rather than transient reinvigoration of exhausted cells, consistent with durable immune reprogramming following localized genome editing. These findings suggest that spatial confinement of genome editing preserves antiviral immune amplification within the tumor while minimizing systemic immune perturbation. Synergy with systemic CTLA-4 blockade further illustrates how localized checkpoint editing can integrate with systemic immunomodulation across spatial scales without overt toxicity.

Building on our prior demonstration of magnetic control of viral delivery, the present study extends this framework to a genome-editing context that integrates spatial gating with programmable checkpoint disruption and immune remodeling. The modular architecture of baculovirus permits incorporation of additional guide RNAs, enabling multiplexed editing within defined tumor regions. Intratumoral viral delivery has been demonstrated across diverse anatomical sites^31^, and magnetic targeting approaches have been applied in preclinical deep-tissue settings^32, 33^, supporting translational feasibility beyond superficial tumors. Nonetheless, local administration and external field application impose practical constraints that will require optimization of injection strategies and magnetic field geometries for clinical deployment.

Together, these findings establish MBV as a controllable genome-editing framework that integrates spatial regulation with endogenous immune activation to enable localized combination immunotherapy. By coupling physical gating with biologically encoded immune signaling, this strategy emphasizes architectural control rather than systemic escalation. More broadly, this work illustrates how external physical inputs can be integrated with intrinsic biological regulatory circuits to achieve spatially regulated genome editing in immunologically active tissue environments.

## Online methods

### Materials

Iron(III) acetylacetonate (Fe(acac)3, 99.9%), oleic acid (90%), oleylamine (70%), 1,2-tetradecanediol (90%), benzyl ether (99%), chloroform (99%), toluene (99.9%), DMSO (99.9%), ferrozine (97%), were purchased from Sigma-Aldrich. 1,2-Distearoyl-sn-glycero-3-phosphoethanolamine-N-[methoxy (polyethylene glycol)-2000] (DSPE-mPEG_2000_) and 1,2-distearoyl-sn-glycero-3-phosphoethanolamine-N-[maleimide(polyethylene glycol)-2000] (DSPE-PEG_2000_-maleimide) were purchased from Avanti Polar Lipids. Cysteine-terminated TAT peptides (CGYGRKKRRQRRR) were synthesized by Genscript.

Bac-to-Bac baculovirus expression system and Cellfectin II was purchased from Thermo Fisher Scientific. BacPAK qPCR titration kit, NucleoMag^®^ NGS Clean-up and Size Select kit, PrimeScript RT Master Kit, and TB Green Premix Ex Taq Master Mix were purchased from Takara Bio. Plasmids pX330 (catalog no. 42230), pCMV-GFP (catalog no. 11153), and pCMV-iRFP (catalog no. 45457) were obtained from Addgene. All gRNAs (Supplementary Table S1) and PCR primers (Supplementary Table S2, S3, and S4) were synthesized by Eurofins. xGen^™^ UDI Primers for NGS library preparation were purchased from Integrated DNA Technologies. Quick-DNA^™^ MicroPrep kit and Quick-RNA Miniprep Kit were purchased from ZYMO Research. jetOPTIMUS^®^ transfection reagent was purchased from Polyplus.

Enzymes, buffers, and kits used for molecular cloning, including Q5^®^ Hot Start High-Fidelity 2× Master Mix, PCR & DNA Cleanup Kit, DNA Gel Extraction Kit, Quick CIP, T4 Polynucleotide Kinase, T4 DNA Ligase Reaction Buffer, BbsI-HF, T7 endonuclease I, and Quick Ligation Kit, were purchased from New England Biolabs. 10-beta competent E. coli cells were also obtained from New England Biolabs.

All antibodies are listed in Supplementary Table S5.

### Production of baculoviral vectors

Recombinant baculovirus vectors were generated using the Bac-to-Bac baculovirus expression system. Briefly, the expression cassettes of Cas9, gRNA, and reporter genes were inserted into the pFastBac vector and transformed into DH10Bac competent cells (Supplementary Fig. S2). The recombinant bacmids containing virus genome and the expression cassettes were extracted using the PureLink HiPure Plasmid Miniprep Kit and transfected into Sf9 insect cells using Cellfectin II according to the manufacturer’s protocol. The insect cell culture medium containing the budded viruses was centrifuged and filtered to remove cell debris using Bottle Top filters (0.45 μm; Thermo Fisher Scientific). The collected recombinant baculoviral vector was amplified in Sf9 cells for two more passages. Baculoviral stocks at passage 3 was used for all experiments. Viral particles were concentrated and purified by filtration through a 0.45 μm Bottle Top filter followed by centrifugation at 6,000 g for 3 h at 4 °C. The resulting viral pellets were resuspended in PBS and stored at 4 °C until use. Baculoviral titers were quantified using the BacPAK qPCR titration kit.

### Synthesis of magnetic nanoparticles

Magnetic nanoparticles (MNPs) were synthesized using a two-step method adapted from previously reported methods^26^. Briefly, magnetite (Fe_3_O_4_) nanocrystals were prepared by thermal decomposition of iron(III) acetylacetonate (Fe(acac)_3_) in benzyl ether in the presence of oleic acid, oleylamine, and 1,2-hexadecanediol. To render the nanocrystals water-dispersible, they were coated with a mixture of DSPE-mPEG_2000_ and DSPE-PEG_2000_-maleimide at a molar ratio of 19:1 using a dual-solvent exchange method^34^. For peptide conjugation, freshly coated MNPs were incubated with cysteine-terminated TAT peptides at a molar ratio of 1:200 in 0.25× PBS overnight at room temperature. Unconjugated peptides were removed by ultracentrifugation at 80,000 g for 35 min at 4°C. The nanoparticles were washed, sterilized by filtration through a 0.22 μm syringe filter, and stored at 4°C until use.

Magnetite nanocrystal size and size distribution of coated MNPs were characterized by transmission electron microscopy (TEM) and dynamic light scattering (DLS). Magnetization curve of nanocrystals was measured at 300K using a superconducting quantum interference device (Quantum Design MPMS). Particle concentrations were quantified using a Ferrozine assay. Successful surface conjugation TAT peptides was confirmed by gel-shift assay (Supplementary Fig. S1).

### Cell culture

MC38 murine colon adenocarcinoma cells were obtained from Kerafast, and Sf9 insect cells were obtained from Thermo Fisher Scientific. All cell lines were cultured according to the manufacturers’ recommended protocols.

### In vitro BV transduction

To evaluate the efficiency and temporal dynamics of BV transduction, MC38 cells were seeded at a density of 4 × 10^4^ cells per well in 24-well plates and allowed to adhere overnight. Cells were then incubated with DMEM/F12 containing BV-eGFP at varying multiplicities of infection (MOIs) for 24 h, after which the medium was replaced with fresh DMEM/F12. eGFP expression in MC38 cells were quantified by flow cytometry at the indicated time points.

To assess magnetic activation and complement-mediated inhibition of BV transduction, MC38 cells seeded in 24-well plates were incubated with BV-eGFP in DMEM/F12, BV-eGFP in DMEM/F12 supplemented with 50% adult mouse serum (AMS), or MBV-eGFP in DMEM/F12 supplemented with 50% AMS. For cells treated with MBV-eGFP, culture plates were placed on a custom-designed magnetic plate for 0.5 h to enable magnetic activation (Supplementary Fig. S3). Cells were subsequently washed and maintained in DMEM/F12. eGFP expression in MC38 cells were assessed at 24 h by fluorescence microscopy and flow cytometry. To evaluate magnetic activation of *Pdl1* disruption under complement pressure, cells were treated with BV-*Pdl1* or MBV-*Pdl1* as described above. eGFP and PD-L1 expression were quantified by flow cytometry at 24 and 48 h post-treatment, respectively.

### Design of sgRNAs for *Pdl1* disruption

Six candidate single-guide RNAs (sgRNAs) targeting early exons or key structural regions of *Pdl1* were designed using a bioinformatics pipeline incorporating the Vienna Bioactivity CRISPR score (Supplementary Table S1)^27^. Potential on- and off- target sites were predicted using the CRISPRO web tool (http://crispor.gi.ucsc.edu/crispor.py)^35^, and the top four predicted off-target loci for each sgRNA were selected for downstream next generation sequencing (NGS) analysis.

MC38 cells were transfected with pX330-sgRNA plasmid using the jetOPTIMUS transfection reagent. Genomic DNA was harvested 48 h post-transfection using the Quick-DNA^™^ MicroPrep kit. Genomic regions encompassing CRISPR-Cas9 cleavage sites were amplified by PCR using Q5 Hot Start High-Fidelity 2× Master Mix under the following conditions: initial denaturation at 98 °C for 30 s; 34 cycles of 98 °C for 10 s, 68 °C for 15 s, and 72 °C for 30 s, followed by a final extension at 72°C for 2 min. For T7 endonuclease I (T7E1) assays, mismatched heteroduplex DNA was generated by denaturing and reannealing 1 μg of purified PCR product, followed by digestion with T7 endonuclease I at 37 °C for 1 h. Digestion products were resolved on 2% agarose gels. Gene-modification efficiency was calculated using the formula: $\%\;\text{Gene}\;\text{modification}=100*(1-\sqrt{1-\text{fraction}\;\text{cleaved}})$.

For targeted NGS indel analysis, genomic regions spanning CRISPR-Cas9 cleavage sites were amplified by PCR. PCR amplicons were purified using the NucleoMag^®^ NGS Clean-up and Size Select kit and subsequently indexed using xGen^™^ UDI 10-nt Primer Plates 1–4 (Integrated DNA Technologies) through a second PCR step (98 °C for 30 s; 30 cycles of 98 °C for 10 s, 64 °C for 15 s, and 72 °C for 30 s; final extension at 72 °C for 2 min). Indexed libraries were purified, verified by 1% agarose gel electrophoresis, and quantified using a Qubit Fluorometer (Thermo Fisher Scientific). Sequencing was performed on an Illumina MiSeq platform. Indel frequencies and editing profiles were analyzed using CRISPResso2 (http://crispresso2.Pinellolab.org)^36^.

### In vitro transcriptomic analysis of BV transduction

MC38 cells were transduced with BV-*Pdl1*, MBV-Cas9, or MBV-*Pdl1* for 48 h. Total RNA was isolated using the Quick-RNA Miniprep Kit. Gene-expression profiling was performed using the nCounter^®^ Mouse Tumor Signaling 360^™^ Panel. Data normalization, differential gene-expression analysis, and pathway enrichment were conducted using the nSolver Analysis Software (v4.0). *P* values in differential gene-expression analysis were adjusted using the Benjamini and Yekutieli method for multiple testing correction.

### In vitro characterization of BV-induced immune responses

BV-induced immune responses were evaluated using coculture systems consisting of BV-transduced MC38 cells with either bone marrow-derived dendritic cells (BMDCs) or splenocytes.

To generate BMDC, bone marrow cells were harvested from female C57BL/6 mice and cultured in RPMI-1640 medium supplemented with 10% fetal bovine serum (FBS), 100 U/mL penicillin, 100 μg/mL streptomycin, 1000 U/mL granulocyte-macrophage colony-stimulating factor (GM-CSF), and 800 U/mL interleukin-4 (IL-4) for 3 days. MC38 cells were transduced with BV-*Pdl1* or BV-Cas9 for 24h, after which they were seeded at a density of 2000 cells per well in 96-well plates and allow to adhere overnight. BMDCs were then added to MC38 cells at a ratio of 2:1 (BMDC:MC38). LPS (100 ng/mL) – treated BMDCs were used as a positive control. After an additional 24 h of coculture, total RNA was extracted using the Quick-RNA Miniprep Kit. RNA was reverse transcribed into cDNA using the PrimeScript RT Master Kit. Gene expression was quantified by real-time quantitative PCR (RT-qPCR) using TB Green Premix Ex Taq Master Mix in a Bio-Rad CFX96 real-time PCR system. Relative gene expression was calculated using the 2^−ΔΔCt^ method.

In parallel experiments, splenocytes were isolated from the spleens of female C57BL/6 mice and cultured in RPMI-1640 medium supplemented with 10% FBS, 100 U/mL penicillin, and 100 μg/mL streptomycin. MC38 cells were transduced with BV for 24 h and subsequently seeded at 2000 cells per well in 96-well plates overnight. Splenocytes were then added at a ratio of 5:1 (splenocytes:MC38). LPS treated splenocytes were used as a positive control. After 24 h of coculture, cytotoxicity was assessed using a Cell Counting Kit-8 (CCK-8) assay. Gene expression was quantified by RT-qPCR as described above.

### Design of the magnetic field for intratumoral BV activation.

The magnetic apparatus used for intratumoral BV activation was designed based on magnet availability, tumor dimensions, and simulated magnetic force distribution. The magnetic force exerted on MNPs was calculated using the equation^37^:

F=μ(m·∇)H

where *μ* denotes the magnetic permeability of the surrounding tissue, ***m*** is the magnetic moment of the MNPs, and ***H*** represents the applied magnetic field.

Based on the numerical simulation, four cylindrical NdFeB magnets (N52 grade; K&J Magnetics) were arranged in an alternating polarity configuration to maximize the magnetic field gradient (Supplementary Fig. S9). A custom 3D-printed animal bed was used to reproducibly position tumors at the center of the magnet array. The resulting magnetic field and force distributions were simulated using a MATLAB program developed in-house.

### Magnetically activated *Pdl1* gene disruption in vivo

All animal studies were approved by the Institutional Animal Care and Use Committee at the University of Kentucky. Female C57BL/6 mice (4–5 weeks old) were purchased from Charles River Laboratories. To establish the syngeneic mouse tumor model, mice received a subcutaneous injection of 5×10^5^ MC38 cells suspended in 100 μL of PBS into the right flank. Tumor volume and body weight were monitored every 3 days. Tumor volume was calculated using the formula V = 1/2 × L × W^2^, where L and W denote tumor length and width, respectively. Treatments were initiated when tumors reached approximately 50 mm^3^. Mouse was euthanized when tumor volume exceeded 1000 mm^3^ in accordance with human endpoint guidelines.

In monotherapy studies, mice were randomized into five groups receiving PBS, MNP alone, BV-*Pdl1* alone, MBV-*Pdl1*, or systemic anti-PD-L1 antibody (aPD-L1). MBV-*Pdl1* was prepared by mixing 1.5×10^7^ pfu of BV-*Pdl1* with 5 μg of MNP-TAT in 20 μL of PBS. Mice received intratumoral infusion of MBV-*Pdl1* every 3 days for a total of four doses. The injectate was delivered using a fanning technique, in which the needle was partially withdrawn and redirected to facilitate intratumoral distribution. The injection volume was fixed at 0.3 × the tumor volume while maintaining constant vector concentration, and the solutions were infused over approximately 5 min using a syringe pump. For mice treated with MNP alone or BV-*Pdl1* alone, injected doses were matched to the corresponding components in the MBV-*Pdl1* formulation. In the MNP alone or MBV-*Pdl1* groups, mice were positioned laterally on a 3D-printed animal bed immediately following infusion, with tumors aligned within the magnetic field and maintained under anesthesia for 1 h (Supplementary Fig. S9). Systemic aPD-L1 was administered intraperitoneally at a dose of 200 μg in 100 μL PBS on the same dosing schedule.

For combination therapy studies, mice were randomized into four groups receiving PBS, systemic aCTLA-4, systemic aCTLA-4 combined with aPD-L1, or MBV-*Pdl1* combined with systemic aCTLA-4, administered every 3 days for a total of four doses. In the aCTLA-4 group, mice received intraperitoneal injection of 200 μg (day 0) or 100 μg (day 3, 6 and 9) of aCTLA-4 in 100 μL PBS^38^. In the aCTLA-4 plus aPD-L1 group, mice received intraperitoneal injection of aCTLA-4 as above and 100 μg aPD-L1 in 100 μL PBS per treatment. In the MBV-*Pdl1* plus aCTLA-4 group, MBV-*Pdl1* was administered intratumorally as described above, followed by intraperitoneal injection of aCTLA-4 as described above.

### Evaluation of in vivo distribution of MBV

To assess the intratumoral distribution of MBV following infusion, tumor-bearing mice received a single intratumoral injection of BV or MBV as described above. In vivo magnetic resonance imaging (MRI) was performed using a 7 T small animal MRI system (PharmaScan, Bruker) equipped with a 38-mm surface coil. Tumors were imaged using a spin-echo sequence with the following parameters: repetition time (TR) = 2,000 ms; echo time (TE) incremented from 12 ms to 144 ms in 12 ms steps; matrix size = 256×256; and field of view (FOV) = 30 mm. T_2_ relaxation maps were generated using a custom MATLAB script.

Following MRI, mice were euthanized, and tumors were harvested, cryosectioned, and stained with Prussian blue to visualize intratumoral MNP distribution.

To assess off-target activities of MBV-*Pdl1* in normal tissues, tumor-bearing mice were treated with PBS or MBV-*Pdl1*-eGFP. To minimize underestimation of off-target events resulting from immune clearance of edited cells, MBV-*Pdl1*-eGFP was administered once at a fourfold dose. 24 h after treatment, mice were euthanized and tumors as well as major organs (heart, kidney, lung, spleen, and liver) were harvested. Each tumor was cut into 4 fragments. Tumor fragments and organs were enzymatically dissociated using an enzyme cocktail (20 μg/mL DNase I, 2 mg/mL Collagenase Type IV, and 0.5 mg/mL Hyaluronidase Type 1-S). Cells were stained with APC-conjugated rat anti-mouse CD45 antibody, PE/Cyanine-conjugated rat anti-mouse/human CD44 antibody, Alexa Fluor 647-conjugated rat anti-mouse/rat/human ER-TR7 antibody and analyzed by flow cytometry.

To assess the *Pdl1* disruption efficiency in the whole tumor, 4 more tumors were harvested 24h after a single injection of MBV-*Pdl1* and minced into 10–12 pieces. Genomic DNA was extracted from mechanically homogenized tissue with a Quick-DNA MicroPrep kit and subjected to targeted NGS amplicon sequencing.

### Immunofluorescence staining

To assess PD-L1 expression and dendritic cell and T-cell infiltration, mice were euthanized 3 days after completion of four treatment cycles, and tumors were harvested, fixed in 4% paraformaldehyde on ice for 2 h, and cryosectioned. Tissue sections were treated with 0.3% H_2_O_2_ for 10 min at room temperature before staining. For PD-L1 detection, sections were incubated with PE-conjugated rat anti-mouse PD-L1 antibody. For visualization of CD3^+^CD8^+^ T cells, sections were blocked with 5% goat serum for 1 h, and incubated with rabbit anti-mouse CD8α antibody for 1 h at room temperature, followed by incubation with Alexa Fluor 594-conjugated rat anti-mouse CD3 antibody and Alexa Fluor 488-conjugated goat anti-rabbit IgG antibody for an additional 1 h. Sections were counterstained with Hoechst 33342 for 5 min at room temperature and mounted using VectaShield Antifade Mounting Media (Vector Laboratories). Similarly, sections were stained for dendrite cells (CD11c^+^CD86^+^). Fluorescence images were acquired using a Nikon Eclipse Ti2 Inverted fluorescence microscope.

### Single-cell RNA sequencing

Immunological changes induced by MBV-*Pdl1* were characterized by single-cell RNA sequencing (scRNA-seq) of tumor-infiltrating immune cells isolated from 3 PBS-treated and 3 MBV-*Pdl1*-treated tumors. Two days after the fourth treatment with PBS or MBV-*Pdl1*, mice were euthanized and tumors were harvested for isolation of CD45^+^ cells following the 10× Genomics Cell Preparation protocol. Tumors were minced into 2–4 mm^3^ fragments and enzymatically dissociated using a tumor dissociation kit (Miltenyi Biotec) for 20 min at 37 °C. Cell suspensions were filtered through a 40-μm cell strainer and centrifuged at 500 g for 7 min at 4 °C. Pellets were resuspended in 2 mL chilled red blood cell lysis buffer and incubated on ice for 3 min, followed by addition of 8 mL RPMI-1640 containing 5% FBS. Cells were filtered through a fresh 40-μm strainer and centrifuged at 500 g for 5 min at 4 °C. Final pellets were resuspended in HBSS supplemented with 5% FBS and labeled with APC-conjugated anti-mouse CD45 antibody for fluorescence-activated cell sorting using a BD FACSymphony^™^ S6 Cell Sorter. For each tumor, up to 10,000 CD45^+^ cells were collected and loaded onto a Chromium Controller (10X Genomics) for single-cell encapsulation. Libraries were prepared according to the manufacturer’s instructions and sequenced on an Illumina HiSeq 2500 platform.

Raw sequencing data were processed using an R-based pipeline. Gene expression libraries were aligned to the mouse reference genome GRCm39 (10X Genomics pre-built reference, GRCm39–2024-A) using Cell Ranger (v8.0.1). Downstream analyses were performed with Seurat (v5.3.0)^39^. Quality control filtering was applied prior to integration. Cells with ≤ 200 detected genes and genes expressed in fewer than three cells were excluded. Cells with total unique molecular identifier (UMI) counts or numbers of detected genes below the 1st percentile or above the 99th percentile of the respective distributions were removed. Additional filtering excluded cells with ≤ 1,000 UMIs, ≤ 500 detected genes, or ≥ 5% mitochondrial transcript content. Putative doublets were identified and removed using scDblFinder (v1.17.0) with default parameters^40^. After quality control and doublet removal, a total of 30,006 cells (6405 (mean) for PBS and 3597 (mean) for MBV-*Pdl1*) were retained for analysis.

Cells from the PBS- and MBV-*Pdl1*-treated tumors were integrated using the Harmony (v1.1.0) to correct for batch effects^41^. Principal component analysis (PCA) was performed, and the first 20 Harmony-corrected principal components were used to construct a shared nearest neighbor (SNN) graph. Unsupervised clustering was performed using the Louvain algorithm with a resolution of 1.4. Uniform Manifold Approximation and Projection (UMAP) was used for visualization. Cell clusters were annotated based on the expression of canonical marker genes. Major immune lineages, including T cells, macrophages, and dendritic cells (DCs), were isolated for independent subclustering analysis. Within each subset, data were renormalized and re-clustered using the same Harmony-based workflow, and subclusters were annotated based on established lineage- and state-specific markers. Comparisons of scRNA-seq-derived cell-state abundances across mice were considered exploratory due to limited biological replicates; conclusions were supported by orthogonal validation assays.

Differential gene expression analysis between clusters or experimental groups was performed using the Wilcoxon rank-sum test. Genes with an adjusted p-value < 0.05 and an absolute log_2_ fold change (log_2_FC) > 0.5 were considered differentially expressed. Gene set enrichment analysis (GSEA) was conducted using the clusterProfiler package (v4.16.0)^42^. Differences in cell-type proportions between experimental groups were assessed using a hypergeometric test.

### Statistical analysis

All statistical analyses were performed using GraphPad Prism. Data were analyzed using two-sided unpaired t-tests (with Welch’s correction when variances were unequal), one-way analysis of variance (ANOVA) with post-hoc Dunnett’s test, or non-parametric tests (Mann–Whitney U test) as appropriate. Tumor volumes at day 9 post-treatment – the last time point at which all mice remained alive prior to control animals reaching humane endpoint criteria – were compared across groups using one-way ANOVA followed by Dunnett’s multiple-comparisons test, with PBS as the control group. Survival data were analyzed using the Log-rank (Mantel-Cox) test. Exact p-values or significance thresholds and sample sizes are reported in the figure legends.

### Schematic illustrations

Schematic illustrations were created with Biorender.com.

## Supplementary Material

Supplement 1

## Figures and Tables

**Fig. 1.**
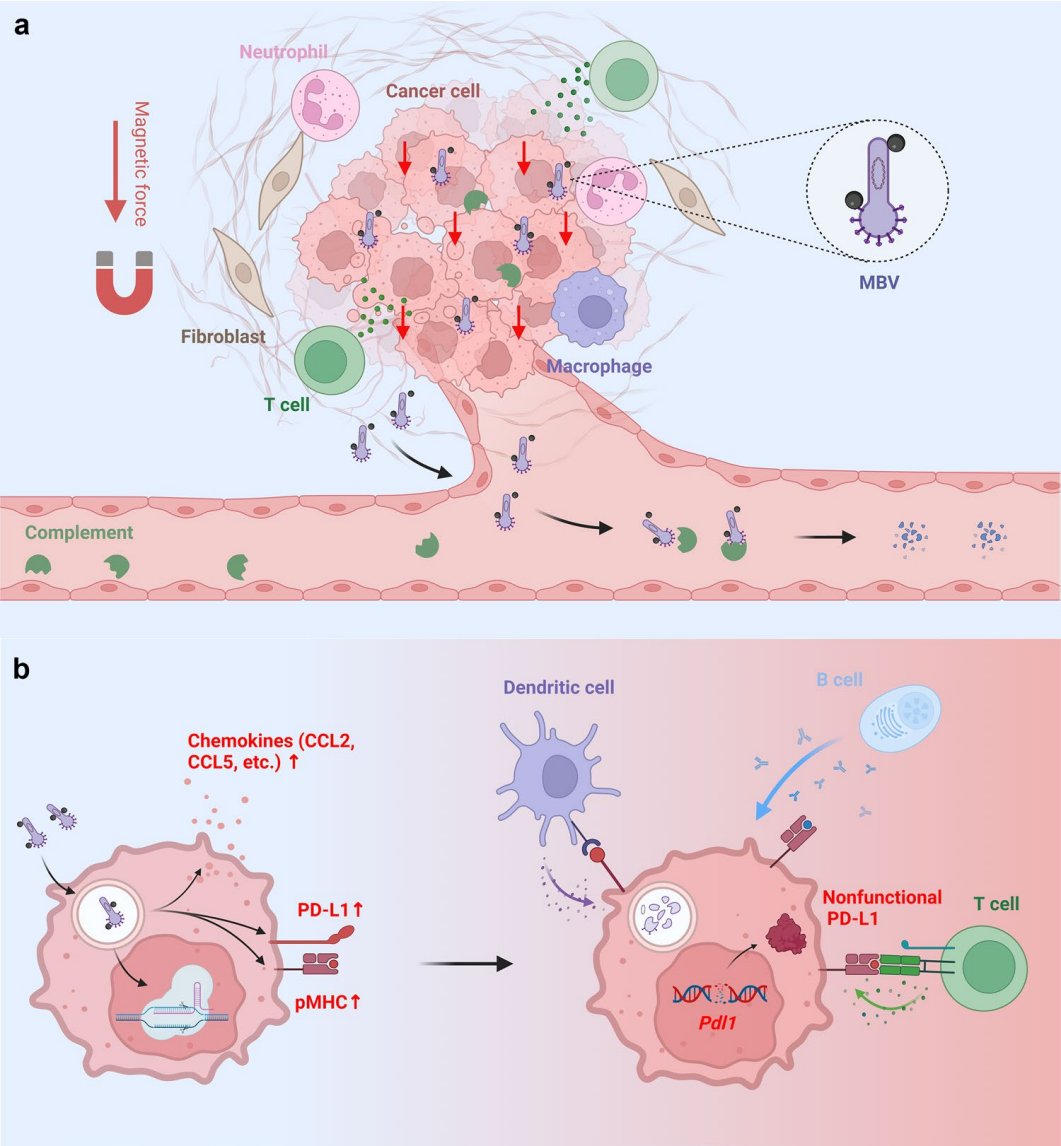
Schematic of MBV-mediated localized genome editing and coordinated anticancer immunity. **a**. MBVs are administered by intratumoral infusion followed by brief magnetic activation. Magnetic activation functions as a physical on-switch that enhances local viral transduction despite complement-mediated inactivation, thereby confining delivery of CRISPR cargo to magnetically targeted tumor regions. MBVs that enter the systemic circulation are inactivated by complement, limiting dissemination and off-target exposure. **b**. MBV transduction engages innate immune sensing pathways, inducing chemokine secretion, enhanced antigen presentation, and PD-L1 upregulation within tumor cells. CRISPR-mediated disruption of *Pdl1* prevents compensatory PD-L1-mediated inhibition, sustaining effector cell activation in the tumor microenvironment. Together, antiviral immune activation and localized *Pdl1* disruption promote spatially confined antitumor immunity.

## Data Availability

Source data underlying the figures are available from the corresponding author upon reasonable request. Raw and processed sequencing data generated in this study will be deposited in a public repository (GEO) prior to publication, and accession numbers will be provided.
