# Magnetically activatable viral genome editing integrates antiviral sensing with checkpoint disruption for localized combination immunotherapy

**DOI:** 10.64898/2026.01.14.699490

**Published:** 2026-03-03

**Authors:** Xiaoyue Yang, Laura Tong, Yidan Pan, Jin Huang, Zhongchao Yi, Daheng He, Jingpeng Liu, Chi Wan, Ying Liang, Sheng Tong

## Abstract

Immune checkpoint blockade can elicit durable tumor regression, yet its therapeutic window is constrained by limited coordination of immunomodulation within tumors and systemic immune perturbation. In vivo genome editing offers a programmable route to durable immunomodulation but has not been integrated with tumor-intrinsic immune regulation in a spatially controlled manner. Here we engineer a magnetically activatable baculoviral genome-editing system that spatially confines CRISPR activity while harnessing intrinsic antiviral sensing to reprogram the tumor microenvironment. Magnetic activation induces localized disruption of *Pdl1* . Baculoviral transduction triggers interferon responses that promote antigen presentation and immune-cell recruitment while simultaneously driving compensatory PD-L1 upregulation that restrains effector function. Local CRISPR-mediated *Pdl1* disruption eliminates this inhibitory feedback, coupling innate immune amplification with sustained checkpoint blockade in a single system. In a syngeneic colon cancer model, magnetically activated vectors restrict *Pdl1* editing to tumors without detectable editing in major organs, suppress tumor growth, and extend survival. Together, these findings define a genome-editing architecture that integrates spatial confinement with durable immune amplification for localized combination immunotherapy.

## Full Text Availability

The license terms selected by the author(s) for this preprint version do not permit archiving in PMC. The full text is available from the preprint server.

