# Supplementary material for: Spatially confined genome editing enables localized combination immunotherapy
Source: bioRxiv. 2026 Feb 18:2026.01.14.699490. Originally published 2026 Jan 15. Preprint. [Version 2] doi: 10.64898/2026.01.14.699490 (PMC12871275; doi:10.64898/2026.01.14.699490)
Supplement: Supplementary file 1 [file media-1.pdf]

Supplementary information

Magnetically activatable insect viral vectors promote anticancer immunity through spatially confined gene disruption

Xiaoyue Yang[1], Laura Tong[2], Yidan Pan[3], Jin Huang[1], Zhongchao Yi[1], Daheng He[1], Jingpeng Liu[1], Chi Wang[1], Ying Liang[4], Sheng Tong[1,]*

[1] F. Joseph Halcomb III, M.D. Department of Biomedical Engineering, University of Kentucky, Lexington, Kentucky 40536, USA

[2] Department of Bioengineering, Stanford University, Stanford, California 94305, USA

[3] Department of Genetics, The University of Texas MD Anderson Cancer Center, Houston, Texas 77030, USA

[4] New York Blood Center, New York, New York 10065, USA

[*] Correspondence: Sheng Tong, sheng.tong@uky.edu

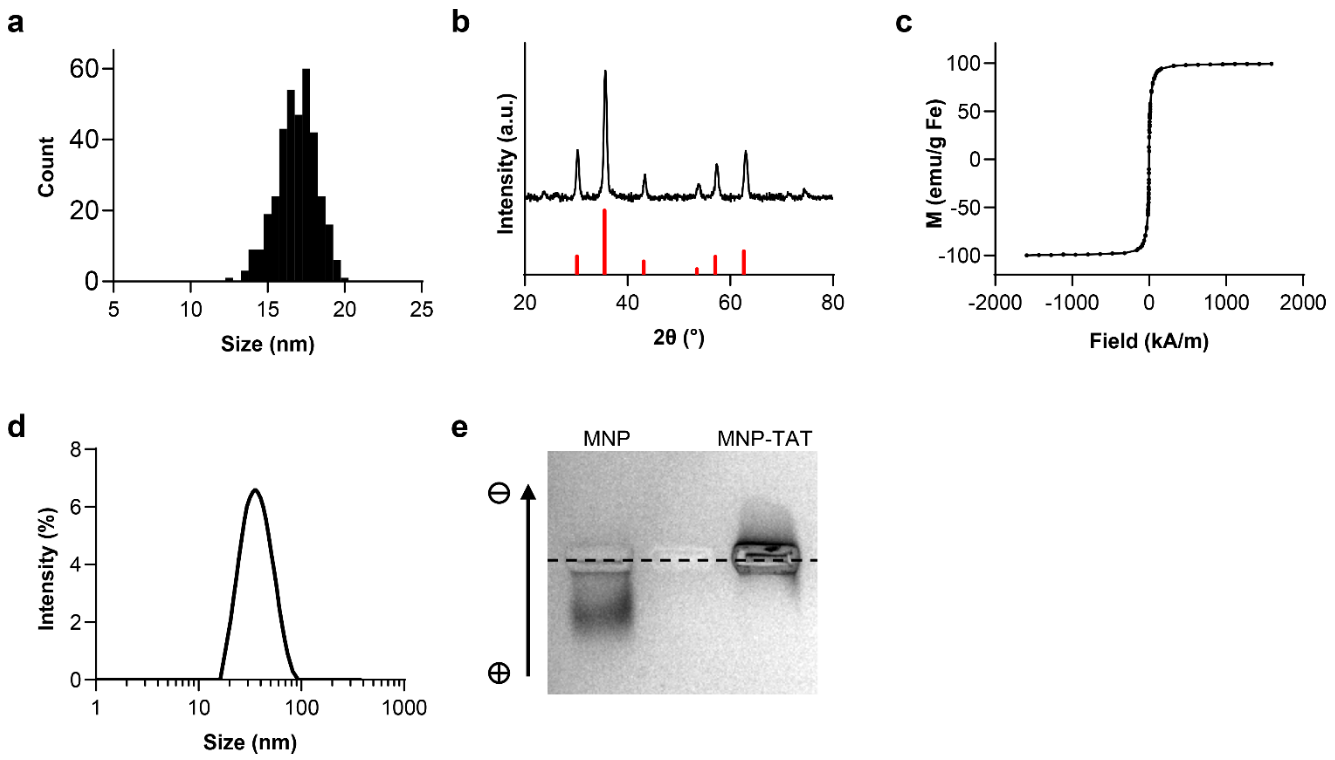

Fig. S1. Characterization of magnetic nanoparticle.

a. Size distribution of iron oxide nanocrystals determined from transmission electron microscopy images (ImageJ analysis). The mean core diameter was 16.9 ± 1.3 nm. **b**. X-ray diffraction (XRD) pattern of iron oxide nanocrystals measured using an X-ray diffractometer (Bruker D8 ADVANCE). Red lines indicate the standard diffraction peaks of magnetite. The measured pattern is consistent with the characteristic cubic spinel structure of magnetite. **c**. Magnetization curves of magnetite nanocrystals measured at 300K using a superconducting quantum interference device (Quantum Design MPMS). **d**. Hydrodynamic size distribution of magnetic nanoparticles (MNPs) measured by dynamic light scattering (DynaPro Nanostar, Wyatt Technology). The mean hydrodynamic diameter was 38.2 ± 0.2 nm with a polydispersity index of 0.231 ± 0.003. **e**. Agarose gel electrophoresis of MNPs with and without TAT peptide conjugation in Tris-acetate EDTA (TAE) buffer (pH8.0). The dashed line indicates the well position. Maleimide-functionalized MNPs migrated toward the anode due to the negative charge of hydrolyzed maleimide (maleic acid), whereas MNP-TAT exhibited slight migration toward the cathode owing to the net positive charge of the conjugated peptides.

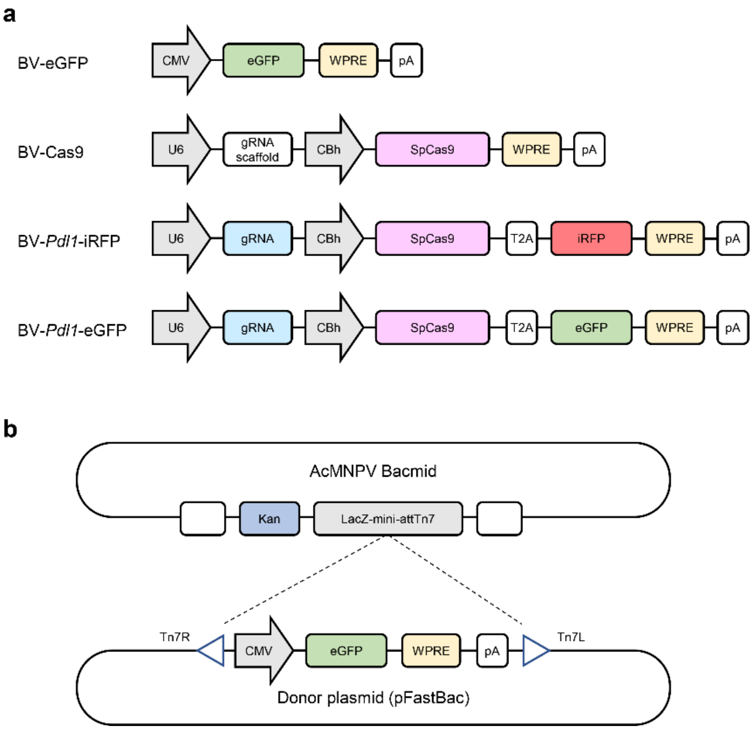

Fig. S2. BV plasmid design.

Baculoviral vectors were generated through three sequential steps. First, expression cassettes encoding Cas9, guide RNAs, and reporter genes (**a**) were assembled using plasmids purchased from Addgene. Second, the expression cassettes were cloned into a donor plasmid (pFastBac), which was subsequently integrated into a bacmid by transformation into DH10Bac competent cells (**b**).

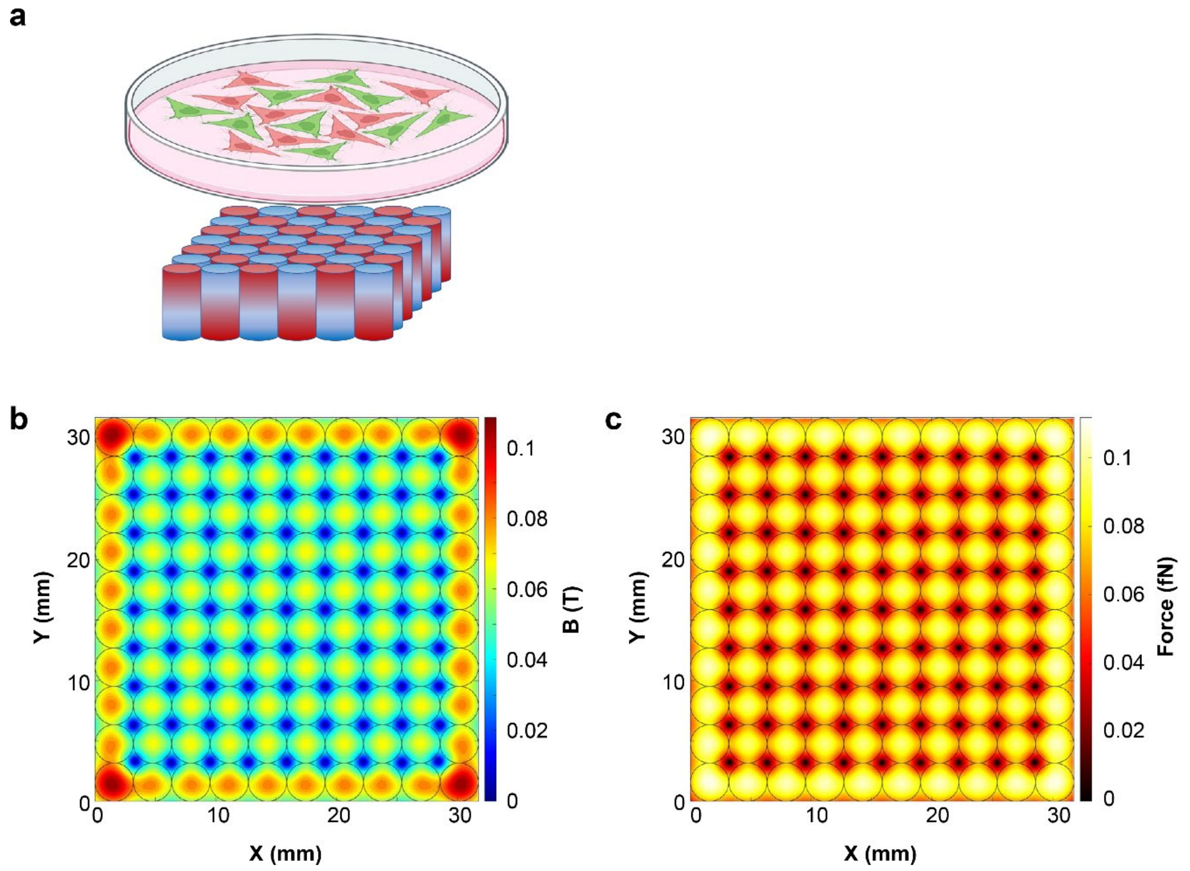

Fig. S3. Magnetic activation in vitro.

a. Schematic of the in vitro magnetic activation setup. A matrix of magnets was placed beneath the cell culture Petri dish. The magnets were N52-grade NdFeB cylindrical magnets with dimensions of 1/8” × 1/4” (diameter × height). Red and blue colors denote the north and south magnetic poles, respectively. The distance between the top surface of the magnets and the cells in the Petri dish was approximately 2 mm. **b**, **c**. Contour plots of the magnetic flux density (**b**) and magnetic force (**c**) at the surface of the Petri dish, calculated for a 10 × 10 magnet matrix. A matrix larger than the culture surface was used in all experiments to avoid edge accumulation of MBV. Black circles denote magnet positions. The magnetic force was calculated for magnetic nanoparticles with a core diameter of 16.9 nm.

a

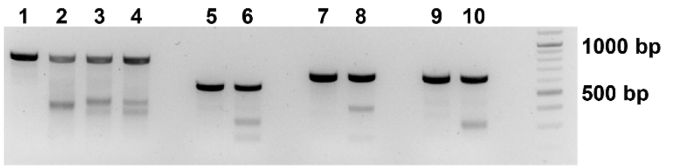

b

Lane	1	2	3	4	5	6	7	8	9	10
Exon	3	3	3	3	5	5	4	4	2	2
Name	CTL	VB1	VB4	VB6	CTL	VB2	CTL	VB3	CTL	VB5
Efficiency (%)	-	24.4	20.5	14.5	-	10.7	-	6.56	-	8.80

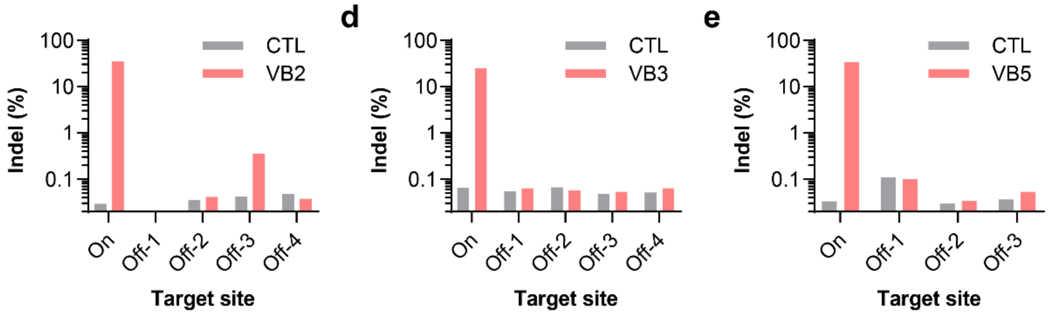

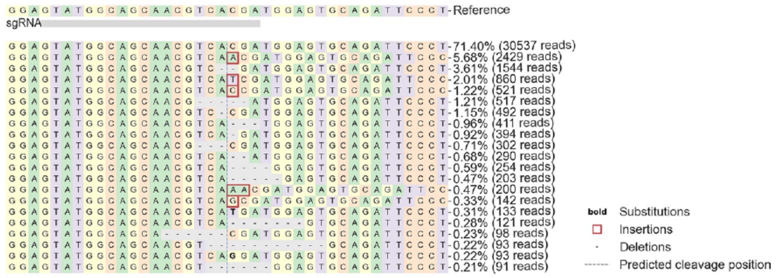

Fig. S4. gRNA screening.

MC38 cells were transfected with pX330 plasmids encoding Cas9 and indicated guide RNAs. gRNA performance was evaluated by assessing on- and off-target efficiencies using the T7 endonuclease I (T7E1) assay and next generation sequencing (NGS), as described in the Methods. **a**. T7E1 analysis of all six candidate gRNAs. **b.** Indel efficiency quantified from the gel shown in **a**. **c-e.** On- and off-target activities of VB2, VB3, and VB5, respectively, following NGS analysis. T7E1 and NGS analysis of gRNAs targeting exon 3 are shown in Fig. 2h-j. **f**. Representative VB1-induced mutation patterns quantified by NGS. VB1 was selected based on its high on-target activity and minimal off-target effects.

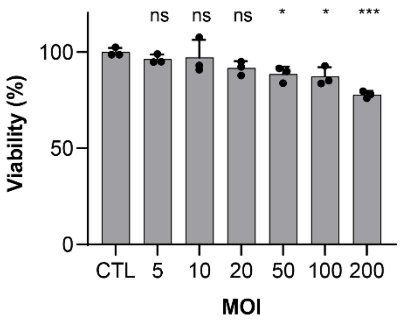

Fig. S5. Cytotoxicity of BV.

To evaluate BV cytotoxicity, MC38 cells were seeded at a density of 2000 cells per well in 96 well plates and allowed to attach overnight. Cells were incubated with BV-*Pdl1* at the indicated multiplicities of infection (MOIs) for 24 hours. Cells were then cultured in fresh medium containing Cell Counting Kit-8 (CCK-8) for 3 h, and absorbance was measured at 450 nm using a microplate reader. Cytotoxicity was calculated as the ratio of absorbance relative to untreated control cells. Data are presented as mean ± s.d. ns, *, and *** denote no significance, $P < 0.05$, and $P < 0.001$ versus control, respectively.

a

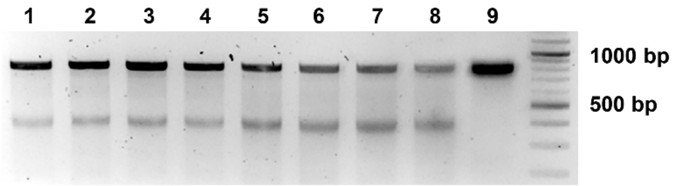

b

Lane	1	2	3	4	5	6	7	8	9
Vector	BV	BV	MBV	MBV	BV	BV	MBV	MBV	Untreated
MOI	20	100	20	100	20	100	20	100	-
Incubation time (h)	24	24	24	24	48	48	48	48	-
Efficiency (%)	9.6	11.0	11.7	11.8	20.4	23.4	26.0	27.0	-

Fig. S6 Efficiency of BV- and MBV-mediated *Pdl1* disruption.

MC38 cells were incubated with *BV-Pdl1* or *MBV-Pdl1* under the indicated conditions. Genomic DNA was harvested using the Quick-DNA^{TM} MicroPrep kit. Genomic regions spanning the CRISPR-Cas9 cleavage sites were amplified by PCR, and gene disruption efficiency was evaluated using the T7 endonuclease (T7E1) assay as described in the Methods. **a**. Gel electrophoresis of T7E1-digested PCR products. **b**. Incubation conditions and indel efficiencies quantified from the gel analysis. MBV showed higher *Pdl1* disruption efficiency relative to BV.

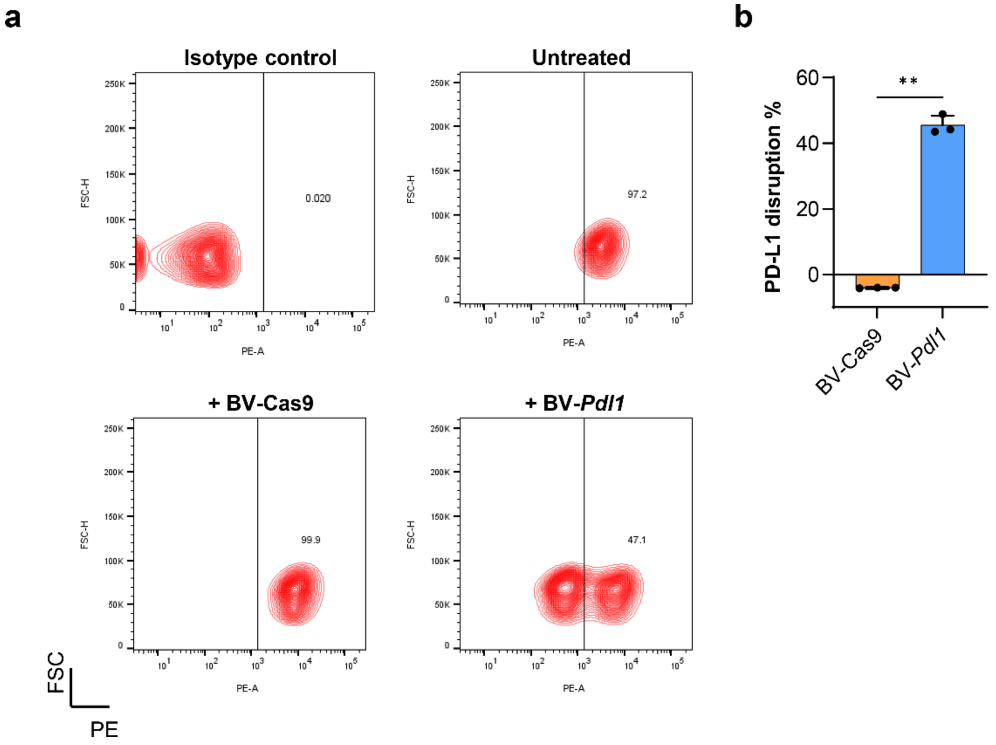

Fig. S7 Effect of BV-Cas9 and BV-*Pdl1* on PD-L1 expression.

To assess the effect of BV-Cas9 and BV-*Pdl1* on PD-L1 expression, MC38 cells were incubated with BV-Cas9 or BV-*Pdl1* at a multiplicity of infection (MOI) of 20 for 24 h. Cells were then cultured in fresh medium for an additional 24 h, after which PD-L1 expression was quantified by flow cytometry. **a**. Representative flow cytometry contour plots. **b**. PD-L1 disruption efficiency calculated as the percentage of PD-L1-negative cells relative to untreated control cells. BV-Cas9 resulted in increased PD-L1 signal, whereas BV-*Pdl1* treatment produced a bimodal PD-L1 distribution, with distinct PD-L1-positive and -negative cell populations. Data are presented as mean ± s.d. ** denotes $P < 0.01$.

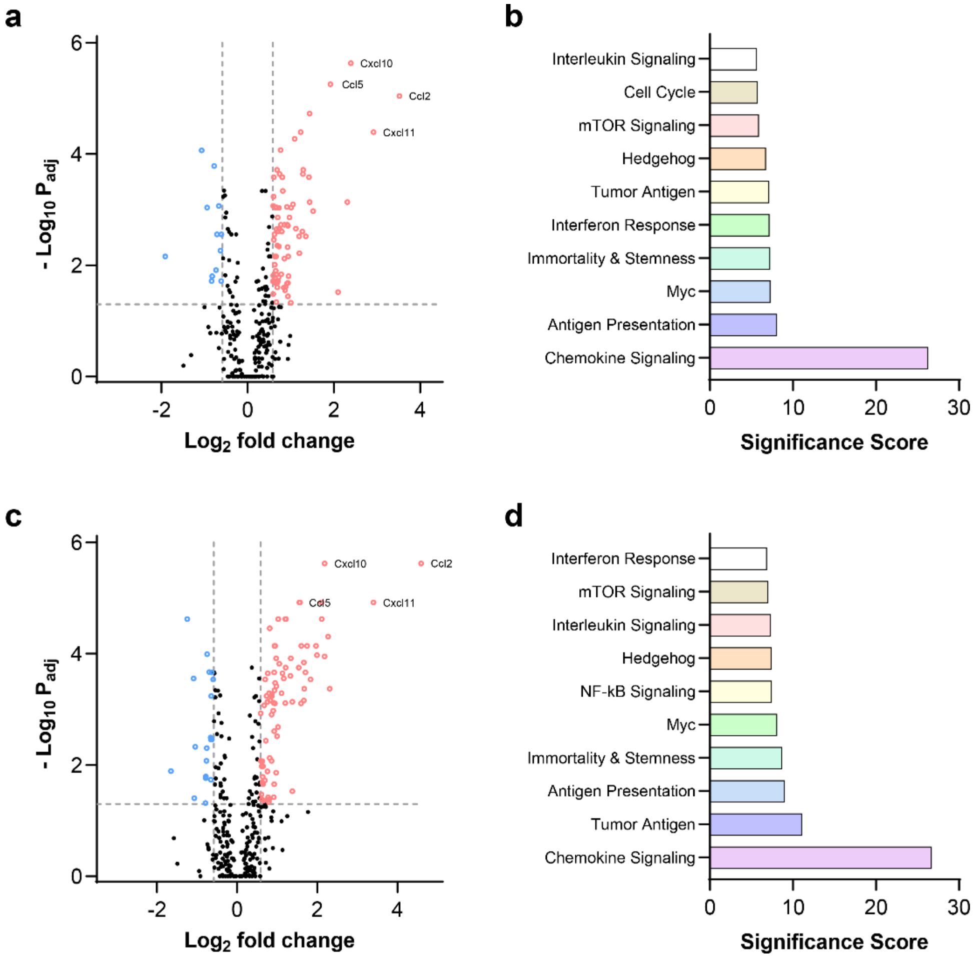

Fig. S8. Gene expression in MC38 cells treated with BV-*Pdl1* and MBV-Cas9.

Transcriptomic responses of MC38 cells treated with BV-*Pdl1* and MBV-Cas9 were analyzed using the nCounter® Tumor Signaling 360 Panel as described in the Methods. All analyses were performed using nSolver Analysis Software (v4.0). **a**. Differential gene expression in MC38 cells treated with BV-*Pdl1* relative to PBS control (n = 3 per group). *P* values were adjusted using the Benjamini and Yekutieli method for multiple testing correction. **b**. Pathway enrichment analysis of BV-*Pdl1*-treated MC38 cells relative to PBS control. **c**. Differential gene expression in MC38 cells treated with MBV-Cas9 relative to PBS control (n = 3 per group). *P* values were adjusted using the Benjamini and Yekutieli method for multiple testing correction. **d**. Pathway enrichment analysis of MBV-Cas9-treated MC38 cells relative to PBS control, revealing pathway signatures overlapping with those induced by MBV-*Pdl1*.

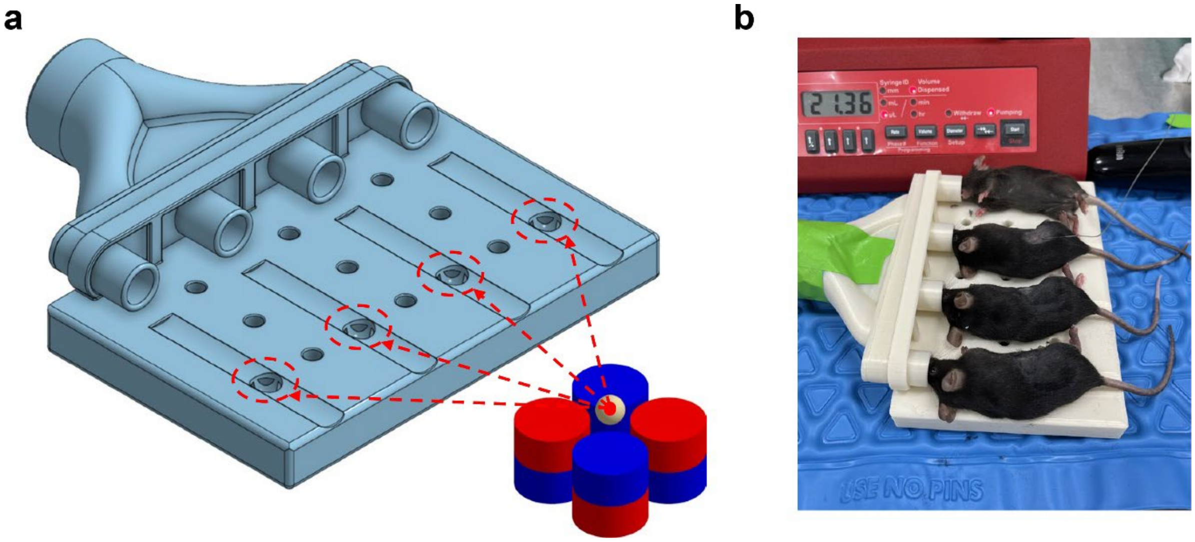

Fig. S9. Schematic of in vivo magnetic activation.

a. Schematic of the custom-designed animal bed. Red dashed circles indicate flank tumor slots. An array of four magnets was installed beneath each tumor slot from the underside of the bed. The magnets were N52-grade NdFeB cylindrical magnets with dimensions of 1/2” × 1/2” (diameter × height). Red and blue colors denote the north and south magnetic poles. **b**. Representative photograph of in vivo magnetic activation. From top to bottom, mouse 1 was undergoing magnetic activation, mouse 2 was receiving MBV infusion controlled by a syringe pump, and mice 3 and 4 were awaiting treatment.

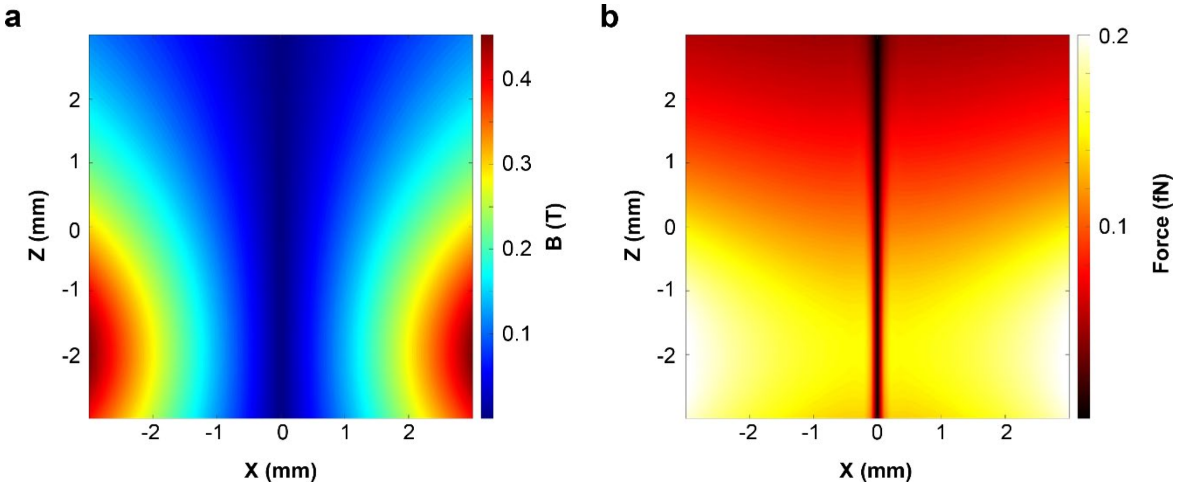

Fig. S10. Simulated magnetic fields on the tumor midplane.

a, **b**. Contour plots of the magnetic flux density (**a**) and magnetic force (**b**) on the central plane of tumors. The geometry of the magnets and tumor is shown in Fig. 4**b**. The magnetic force was calculated for magnetic nanoparticles with a core diameter of 16.9 nm.

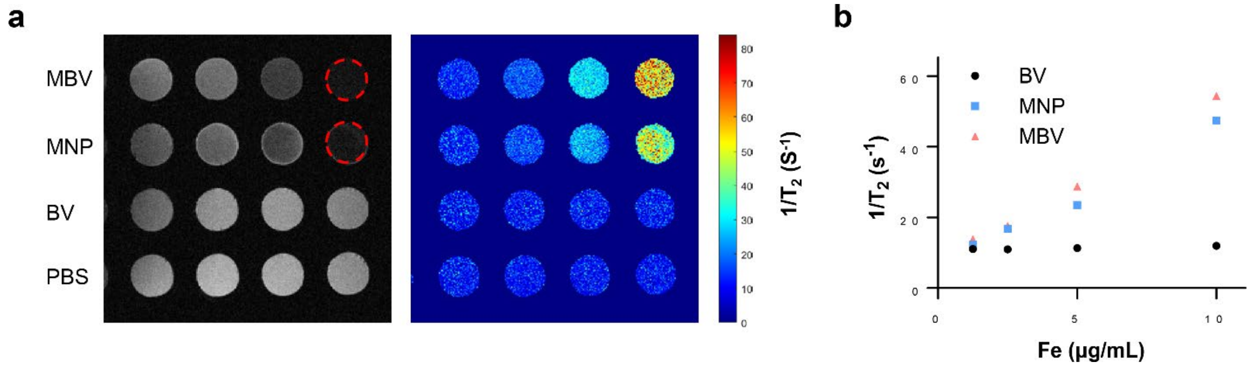

Fig. S11. MRI T_2 relaxivity of MBV.

To evaluate the T_2 contrast properties of MBV, MBV was prepared as described in the Methods. Solutions of MNP, BV, and MBV was loaded into a 3D-printed mold and imaged using a 7 T small animal MRI system (PharmaScan, Bruker) equipped with a 38-mm surface coil. MNP and MBV solutions were matched for iron concentration, and BV and MBV solutions were matched for BV concentration. **a**. T_2-weighted MRI image of MNP, BV, and MBV solutions and the corresponding T_2 map; PBS was used as a reference. Dashed red circles indicate the positions of wells not visible in the image. **b**. T_2 relaxivity of MNP, BV, and MBV calculated from the T_2 map in **a**. At matched iron concentrations, MBV exhibited slightly higher T_2 relaxivity than MNP, consistent with the accumulation of MNP on the BV surface.

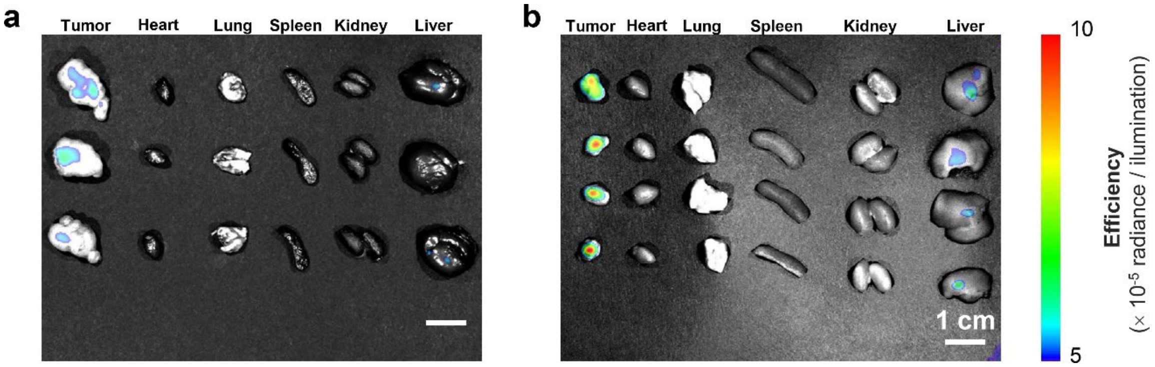

Fig. S12 Ex vivo imaging of BV transduction.

Tumor-bearing mice were treated with PBS (**a**) or MBV-*Pdl1*-iRFP (**b**) as described in the Methods. 48 h after completion of four treatment, mice were euthanized, and tumors and major organs (heart, lung, spleen, kidney, and liver) were collected and imaged (ex/em = 535/590 nm) using an Ami HT small-animal imaging system.

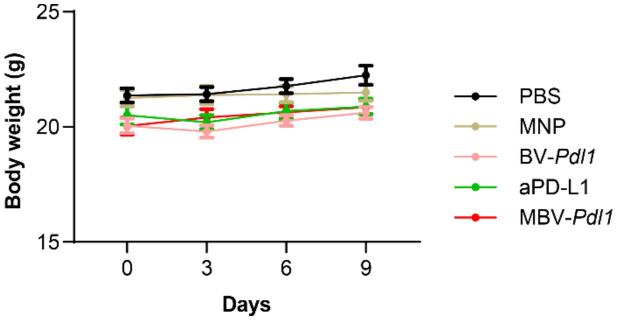

Fig. S13 Body weight monitoring during treatment shown in Fig. 4.

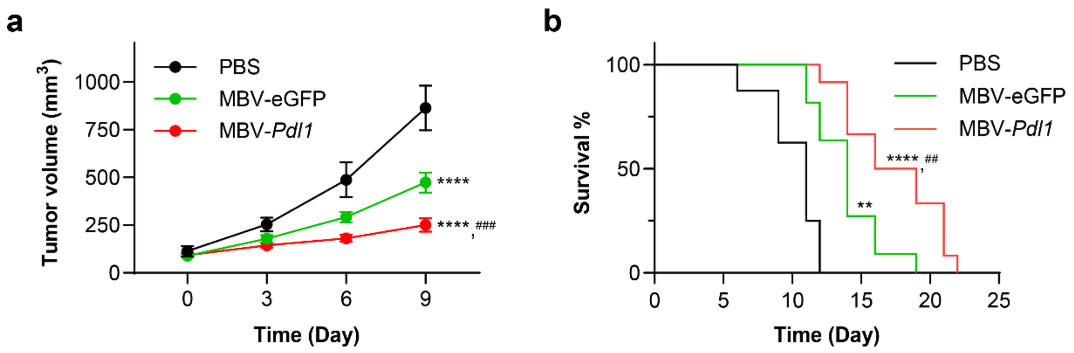

Fig. S14. Effects of MBV-eGFP and MBV-*Pdl1* on tumor growth.

Mice received a subcutaneous injection of 5×10^5 MC38 cells suspended in 100 μL of PBS into the right flank. Treatments were initiated when tumor volumes reached approximately 50 mm^3. Mice were randomized to receive PBS, MBV-eGFP, or MBV-*Pdl1*. MBVs were administrated by intratumoral infusion as described in the Methods, except that the MBV concentration was increased fourfold. **a**, **b**. Tumor volume (**a**) and survival curve (**b**). Data are presented as mean ± s.e.m. Sample sizes were n = 8 (PBS), 11 (MBV-eGFP), and 12 (MBV-*Pdl1*). ** and **** denote $P < 0.01$ and $P < 0.0001$ versus PBS, respectively; ## and ### denote $P < 0.01$ and $P < 0.001$ versus MBV-eGFP, respectively. MBV-*Pdl1* treatment resulted in reduced tumor growth and prolonged survival relative to MBV-eGFP, consistent with a contribution from *Pdl1* disruption.

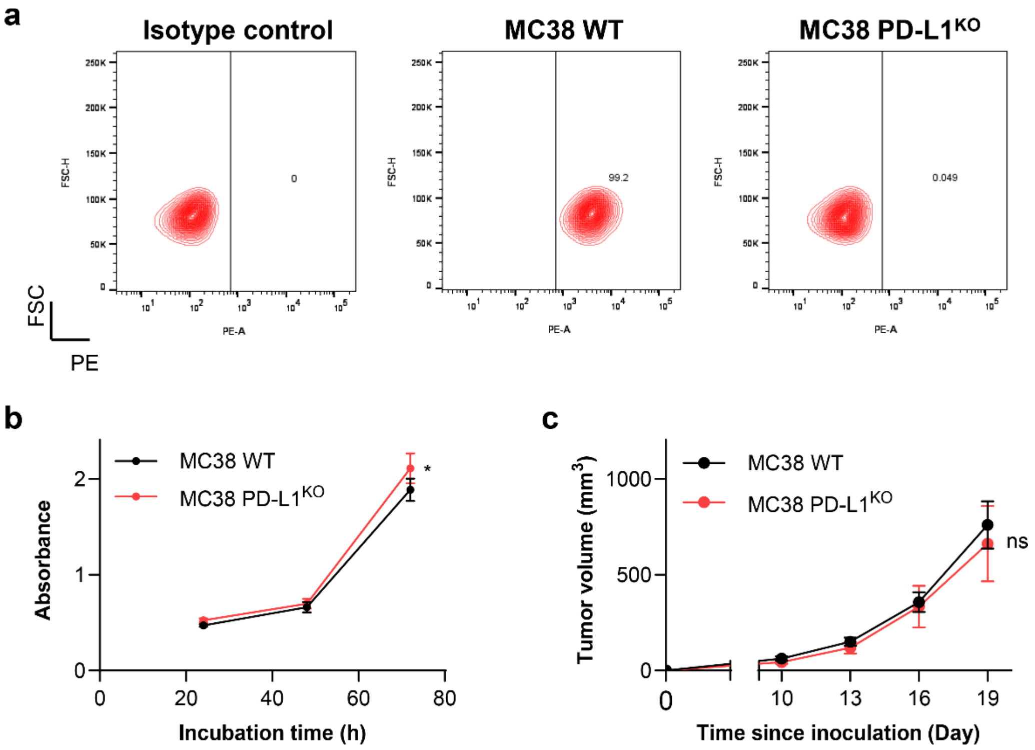

Fig. S15. Tumorigenicity of MC38 WT versus MC38 PD-L1^{KO} cells.

To assess the contribution of PD-L1 expression to tumorigenicity, a monoclonal MC38 line with PD-L1 knockout disruption was generated by transfection with a pX330-*Pdl1* plasmid. **a**. Loss of PD-L1 expression confirmed by flow cytometry. **b**. In vitro cell growth measured by CCK-8 assay. Data are presented as mean ± s.d. (n = 4 per group). * denotes $P < 0.05$ versus MC38 WT. **c**. In vivo tumor growth curves. Data are presented as mean ± s.e.m. Sample sizes were n = 8 (MC38 WT) and n = 7 (MC38 PD-L1^{KO}). ns denotes no significant difference versus MC38 WT. MC38 PD-L1^{KO} cells exhibited a modest reduction in tumorigenicity ($P = 0.11$, two-tailed paired t test).

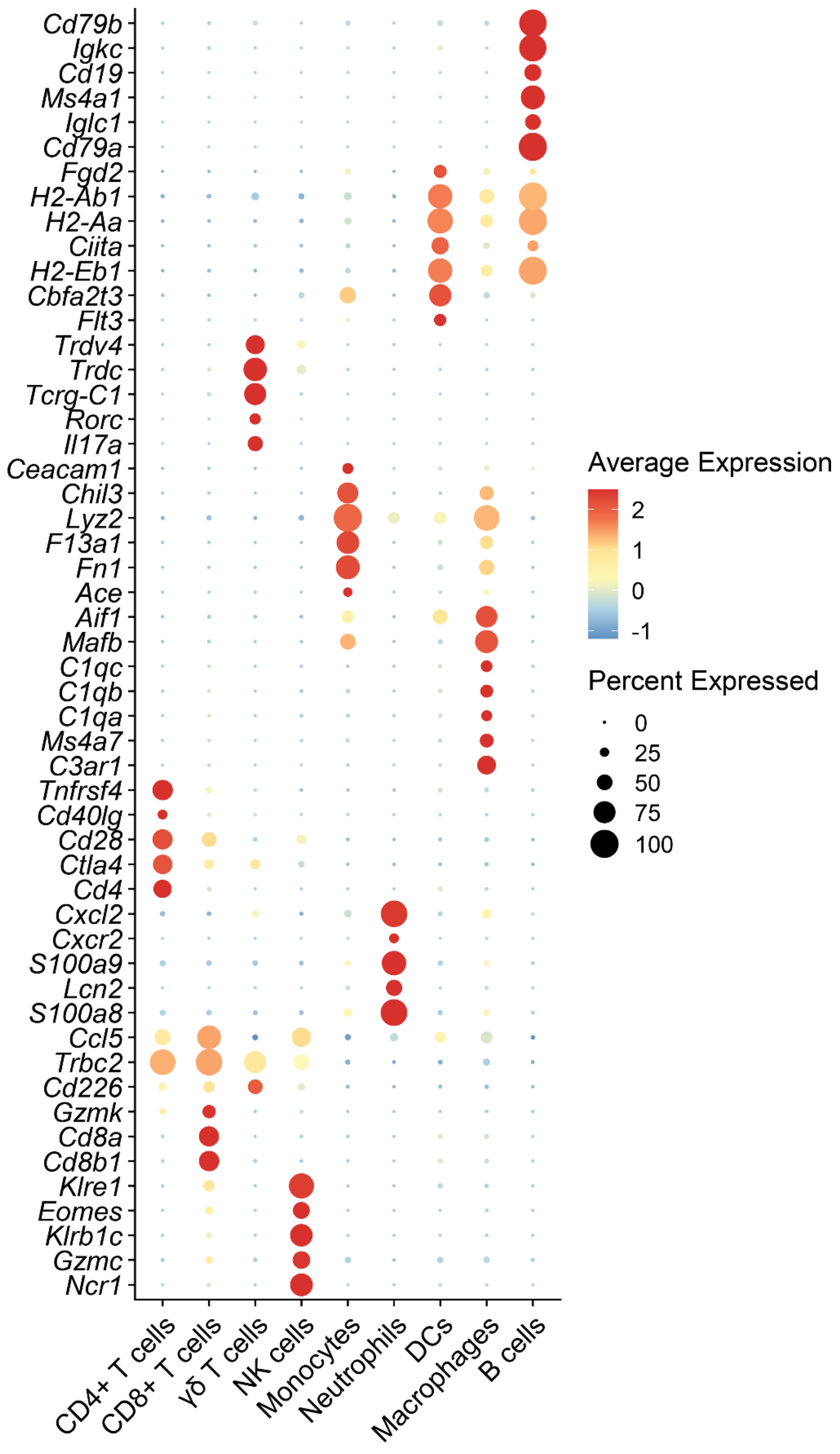

Figure S16 Marker Expression Across Immune Cell Types

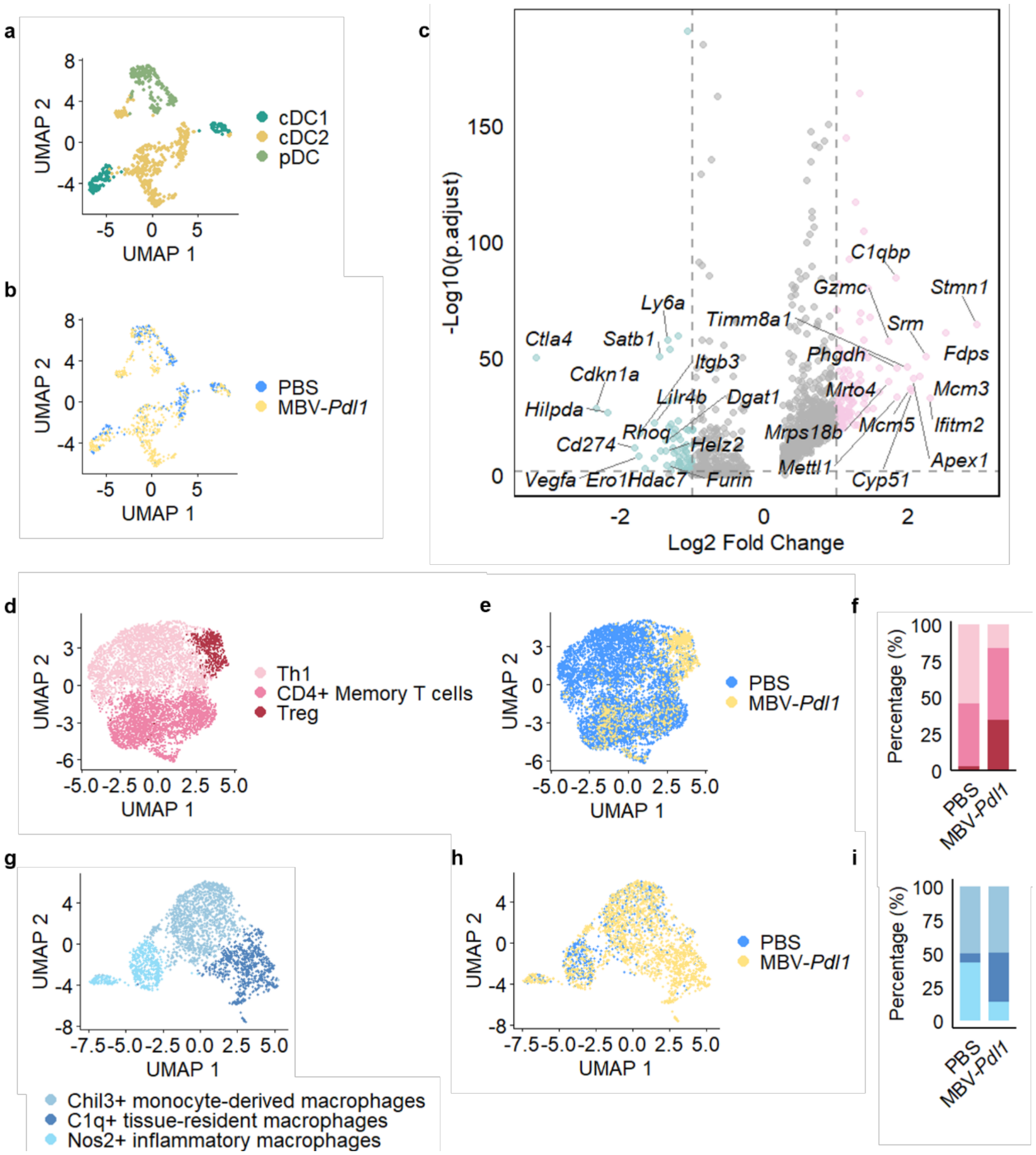

Fig. S17. Single cell RNA sequencing of tumor infiltrating immune cells.

The effects of MBV-*Pdl1* treatment on tumor immune microenvironment was analyzed by single-cell RNA sequencing, in addition to the analyses shown in Fig. 5. **a**. UMAP embedding showing clustering of dendritic cells (DCs). **b**. UMAP showing the distribution of DCs in PBS- and MBV-*Pdl1*-treated tumors. **c**. Volcano plot of differentially expressed genes in NK cells, showing upregulation of proliferation-associated (*Stmn1*, *Mcm3*, *Mcm5*), cytotoxic-associated (*Gzmc*, *Ifitm2*, *Apex1*), and metabolic-associated (*Cyp51*, *Fdps*, *Phgdh*, *Srm*, *Mettl1*) genes, and downregulation of inhibitory/exhaustion-associated (*Ctla4*, *Cd274*, *Satb1*) and stress-adaption-associated (*Cdkn1a*, *Hilpda*, *Vegfa*, *Ero1l*) genes. **d**. UMAP embedding showing clustering of $CD4^+$ T cells. **e**. UMAP showing the distribution of $CD4^+$ T cells in PBS- and MBV-*Pdl1*-treated tumors. **f**. Proportions of $CD4^+$ T cell subtypes in PBS- and MBV-*Pdl1*-treated tumors. **g**. UMAP embedding showing clustering of macrophages. **h**. UMAP showing the

distribution of macrophages in PBS- and MBV-*Pdl1*-treated tumors. **i**. Proportions of macrophage subtypes in PBS- and MBV-*Pdl1*-treated tumors.

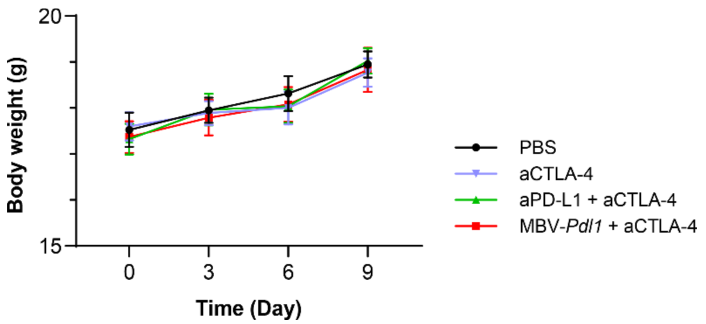

Fig. S18 Body weight monitoring during treatment shown in Fig. 6.

Supplementary Table S1 List of guide RNAs

gRNA	gRNA + NGG (5' - 3')	Exon number	VBC-score
VB1	GTATGGCAGCAACGTCACGATGG	Exon 3	0.75
VB2	GGACCGTGGACACTACAATGAGG	Exon 5	0.757
VB3	GCCAGGGCAAAACCACACAGCGG	Exon 4	0.716
VB4	GGCTCCAAAGGACTTGTACGTGG	Exon 4	0.725
VB5	GCCTGCTGTCACTTGCTACGGGG	Exon 2	0.686
VB6	AGTACACCACTAACGCAAGCAGG	Exon 3	0.701

Supplementary Table S2 List of primers for T7E1 assay

**gRNA**	**Forward** (5' - 3')	**Reverse** (5' - 3')
VB1	AATGAACAACAACCGCCC	CGAACGAATGAACAAACGAG
VB2	CAAGGAAGTTACTGCACTAAGG	GTGTAACTGAAAGCAAGCCC
VB3	GCAGACTAACACTCACTCCC	TCCTATCCAGCCACGAATAC
VB4	AATGAACAACAACCGCCC	CGAACGAATGAACAAACGAG
VB5	CCCATCATACTGACTTCTTTCC	TAGAAGCCAGGTGCAGTAG
VB6	AATGAACAACAACCGCCC	CGAACGAATGAACAAACGAG

Supplementary Table S3 List of primers for NGS

On target

**gRNA**	**Forward** (5' - 3')	**Reverse** (5' - 3')
VB1	ACACGACGCTCTTCCGATCTTCTGTCTTCTGAGGGCTGGT	GACGTGTGCTCTTCCGATCTTAAGGTCCTCCTCTCCTGCC
VB2	ACACGACGCTCTTCCGATCTACCTTCCATCAGCTTCTCTT	GACGTGTGCTCTTCCGATCTGATGTGGGTCTGTTCTTGTC
VB3	ACACGACGCTCTTCCGATCTAGAGGGGATGCTTCTCAATG	GACGTGTGCTCTTCCGATCTTCGGCCAACTACTGCTAAAT
VB4	ACACGACGCTCTTCCGATCTTCTGTCTTCTGAGGGCTGGT	GACGTGTGCTCTTCCGATCTTAAGGTCCTCCTCTCCTGCC
VB5	ACACGACGCTCTTCCGATCTAGACCTCTCTGTGTTTCCCG	GACGTGTGCTCTTCCGATCTCGCTTTATGTGTGCCTAGCA
VB6	ACACGACGCTCTTCCGATCTTATGGCAGCAACGTCACGAT	GACGTGTGCTCTTCCGATCTTGCAGCTTGACGTCTGTGAT

Off target

**gRNA**	**Forward** (5' - 3')	**Reverse** (5' - 3')
VB1-1	ACACGACGCTCTTCCGATCTTCCCGAGCCTTACGTACAGA	GACGTGTGCTCTTCCGATCTGCACGGGTCTCTCCATTCTC
VB1-2	ACACGACGCTCTTCCGATCTTGGCTAACATTTAAAGGCAC	GACGTGTGCTCTTCCGATCTTGGGAAGAAAGTCCAACTCA
VB1-3	ACACGACGCTCTTCCGATCTGTGAGCCACAAGGAAGTATG	GACGTGTGCTCTTCCGATCTCCATCCAACCCACAGAAAAT
VB1-4	ACACGACGCTCTTCCGATCTAGTCATCCTCCACACCCAGC	GACGTGTGCTCTTCCGATCTGCTGCCTCCCAGGTAACCTT
VB2-1	ACACGACGCTCTTCCGATCTTCCACATTGGAGCATCAGCA	GACGTGTGCTCTTCCGATCTAGTGTGTTTTGAAAAGAAGGGAGA
VB2-2	ACACGACGCTCTTCCGATCTTCTTTGGGCCTCTTCAGCAG	GACGTGTGCTCTTCCGATCTCGTCACCCATAATCCCCTGG
VB2-3	ACACGACGCTCTTCCGATCTATATGGGCAACAATTGGACC	GACGTGTGCTCTTCCGATCTCAGCATCACTGTTCAAGACA
VB2-4	ACACGACGCTCTTCCGATCTAGATTTACTACCCCAGAGCC	GACGTGTGCTCTTCCGATCTATGTACAACCATCTGGGACA
VB3-1	ACACGACGCTCTTCCGATCTACCATGGGTTATGATGGCCA	GACGTGTGCTCTTCCGATCTGGAGAACCCATTACGACGGT
VB3-2	ACACGACGCTCTTCCGATCTCTCAGAAGACCACGAGGCAG	GACGTGTGCTCTTCCGATCTGCATCCCTGGGAATATGCCA
VB3-3	ACACGACGCTCTTCCGATCTGCAGTTCACTTCATAGTACATGG	GACGTGTGCTCTTCCGATCTAATGCACGTGACAGAGATGT
VB3-4	ACACGACGCTCTTCCGATCTGCTGGCCTCCATCTCCTGAT	GACGTGTGCTCTTCCGATCTTCTTACAGAGTGGGACCAGCA
VB4-1	ACACGACGCTCTTCCGATCTGCTAGCCATCCCACGGAAAA	GACGTGTGCTCTTCCGATCTGGACGTTCTCTGTCTGCCCT
VB4-2	ACACGACGCTCTTCCGATCTGGGGAATCCTGACCCTGCAA	GACGTGTGCTCTTCCGATCTTCCGCTAGTGGGAATTCAGC
VB4-3	ACACGACGCTCTTCCGATCTGCTTCGAGTGACTCCAGCTT	GACGTGTGCTCTTCCGATCTGGCTAAGGGCAATGGGACAT
VB4-4	ACACGACGCTCTTCCGATCTACAAGCTGCTGAAAACCTGG	GACGTGTGCTCTTCCGATCTCCTCTGAAGAAGGCTCACGA
VB5-1	ACACGACGCTCTTCCGATCTCCTGAACCCACGCCCCATAA	GACGTGTGCTCTTCCGATCTTGTCACCCTTGCCTCTCTTGG
VB5-2	ACACGACGCTCTTCCGATCTCTGAGATCGTCTGCAGACCC	GACGTGTGCTCTTCCGATCTCAGCCCAGGGACAGTATTGG
VB5-3	ACACGACGCTCTTCCGATCTGCAGTTTGTCTGTGTCCTGG	GACGTGTGCTCTTCCGATCTTGACAGGGGTCCTTTTCTCC
VB5-4	ACACGACGCTCTTCCGATCTAACTGTGTGCTGGTGTGACA	GACGTGTGCTCTTCCGATCTTTTTCCTGTGACGGACGTGC
VB6-1	ACACGACGCTCTTCCGATCTCACTTCTCTGCTGGCTGGAA	GACGTGTGCTCTTCCGATCTTGTCAAAGCCATGCTCTCGT
VB6-2	ACACGACGCTCTTCCGATCTACGAGAGGCACATGTTGAGA	GACGTGTGCTCTTCCGATCTACAATGATGCTGCCTTCCTCT
VB6-3	ACACGACGCTCTTCCGATCTGAACTGGCTTGCGTAGACCT	GACGTGTGCTCTTCCGATCTTACCCAGAGCTAGCCTCCTG
VB6-4	ACACGACGCTCTTCCGATCTTCTGCAACCAAACTGGGCTT	GACGTGTGCTCTTCCGATCTGCTAGACTTTGGCAGGAACCT

Supplementary Table S4 List of primers for RT-qPCR

**Gene**	**Forward** (5' - 3')	**Reverse** (5' - 3')
GAPDH	GCATCTTCTTGTGCAGTGCC	ACTGTGCCGTTGAATTTGCC
CD86	ACGGACTTGAACAACCAGACT	CGTCTCCACGGAAACAGCAT
CD40	CTGCTGGTCATTCCTGTCGT	GTTCCAGGGTTCAGACCAGG
IL-12	TAGAGGTGGACTGGACTCCC	GTGAGTGGCTCAGAGTCTCG
TNF-α	CAGTTCTATGGCCCAGACCC	TAGCAAATCGGCTGACGGTG
IFN-α1	AGGACTTTGGATTCCCGCAG	TCATTGAGCTGCTGGTGGAG
IFN-β1	CAACCTCACCTACAGGGCG	CTGTCTGCTGGTGGAGTTCAT
GranB	AGGACTTTGTGCTGACTGCT	TCACATTGACATTGCGCCTG
IFN-γ	CGGCACAGTCATTGAAAGCC	TGCATCCTTTTTCGCCTTGC
IL-2	GAAACTCCCCAGGATGCTCA	AAAGTCCACCACAGTTGCTGA
IL-10	GGTTGCCAAGCCTTATCGGA	ACACCTTGGTCTTGGAGCTTA

Supplementary Table S5. List of antibodies.

**Name**	**Vendor**	**Catalog no.**
*InVivo*MAb anti-mouse PD-L1 (clone 10F.9G2™)	Bio X Cell	BE0101
*InVivo*MAb anti-mouse CTLA-4 (clone 9H10)	Bio X Cell	BE0131
Alexa Fluor® 594-conjugated rat anti-mouse CD3	Biolegend	100240
APC-conjugated rat anti-mouse CD45	Biolegend	103112
PE/Cyanine-conjugated rat anti-mouse CD44	Biolegend	163607
PE-conjugated rat anti-mouse PD-L1	Biolegend	124308
Alexa Fluor® 594-conjugated hamster anti-mouse CD11c	Biolegend	117346
Rat anti-mouse CD86	Abcam	ab119857
Rabbit anti-mouse CD8a	Abcam	ab217344
Alexa Fluor® 555-conjugated goat anti rat antibody	Abcam	ab150158
Alexa Fluor® 488-conjugated goat anti rabbit antibody	Abcam	ab150077
Alexa Fluor® 647-conjugated rat anti-mouse ER-TR7	Santa Cruz Biotechnology	Sc-73355
